# Understanding cell fate decisions with temporal attention

**DOI:** 10.1371/journal.pcbi.1014813

**Published:** 2026-10-12

**Authors:** Florian Bürger, Martim Dias Gomes, Adrián E. Granada, Noémie Moreau, Katarzyna Bozek

**Affiliations:** 1 Institute for Biomedical Informatics, Faculty of Medicine, University of Cologne and University Hospital Cologne, Cologne, Germany; 2 Faculty of Mathematics and Natural Sciences, University of Cologne, Cologne, Germany; 3 Cologne Excellence Cluster on Cellular Stress Responses in Aging-Associated Diseases (CECAD), University of Cologne, Cologne, Germany; 4 Center for Molecular Medicine Cologne (CMMC), University of Cologne and University Hospital Cologne, Cologne, Germany; 5 Granada Lab, Berlin, Germany; Harbin Institute of Technology (Shenzhen), CHINA

## Abstract

Understanding non-genetic determinants of cell fate is critical for developing and improving cancer therapies, as genetically identical cells can exhibit divergent outcomes under the same treatment conditions. In this work, we present a deep learning approach for cell fate prediction from raw long-term live-cell recordings of cancer cell populations under chemotherapeutic treatment. Our Transformer model is trained to predict cell fate directly from raw image sequences, without relying on predefined morphological or molecular features. Beyond classification, we introduce a comprehensive explainability framework for interpreting the temporal and morphological cues guiding the model’s predictions. We demonstrate that prediction of cell outcomes is possible based on the video only, our model achieves balanced accuracy of 0.94 and an F1-score of 0.93. Attention and masking experiments further indicate that the signal predictive of the cell fate is not uniquely located in the final frames of a cell trajectory, as reliable predictions are possible up to 8 h before the event. Our analysis reveals distinct temporal distribution of predictive information in the mitotic and apoptotic sequences, as well as the role of cell morphology and p53 signaling in determining cell outcomes. Together, these findings demonstrate that attention-based temporal models enable accurate cell fate prediction while providing biologically interpretable insights into non-genetic determinants of cellular decision-making.

## Author summary

Cancer treatment often fails because not all cells respond in the same way. Even when exposed to the same drug, some cells die while others survive and continue to grow, which can lead to relapse. Understanding why genetically identical cells make different decisions is therefore an important challenge. In this study, we investigate whether it is possible to predict the fate of individual cells directly from time-lapse microscopy videos. We develop a computational approach that learns from raw image sequences without relying on manually defined features. This allows us to capture subtle changes in cell behavior over time. We find that cell fate can be predicted with high accuracy based on the video data alone, and that informative signals appear well before the final outcome is visible. By analyzing the model, we identify when during a cell’s lifetime predictive information emerges and which aspects of cell behavior are most relevant. Our results show that dynamic patterns in cell behavior contain early indicators of fate decisions, providing new opportunities to better understand variability in treatment response.

## 1 Introduction

A major challenge in cancer treatment is the partial response of tumors to chemotherapeutic drugs. Even when initially efficacious, such treatments often leave behind residual tumor cells, which can subsequently give rise to resistant clones and drive further disease progression. Understanding the mechanisms underlying cellular non-responsiveness to therapy is therefore of critical importance. Individual cells within identical population can exhibit markedly different outcomes under the same therapeutic conditions. While some cells undergo programmed cell death in response to treatment, others survive and continue proliferating, ultimately contributing to therapy resistance and relapse. Genetic factors play a key role in treatment response, however, a broad range of cell-intrinsic factors can lead to heterogeneous responses even among genetically identical cells [1–3]. These cells may differ in their epigenetic, transcriptomic, and proteomic states [4]. Understanding the non-genetic factors that drive divergent outcomes is essential for improving treatment strategies and for gaining insight into fundamental cellular decision-making processes. Previous studies [4–7] have identified multiple molecular factors influencing cell fate following treatment. In addition to markers such as p53, these studies highlight the importance of dynamic cellular properties, including cell-cycle phase and proliferative state, in determining drug susceptibility. Although these studies inspected the link of individual factors to cell fate, a systematic investigation of the predictability of cell fate under chemotherapeutic treatment given various dynamical factors is still lacking.

Deep learning methods provide a powerful means to investigate not only molecular determinants of cell fate, but also morphological and spatial aspects of the cellular environment. Rather than relying on the manual selection and quantification of predefined features [8], deep learning approaches enable direct assessment of cell fate predictability using raw time-lapse microscopy image data. This allows simultaneous capture of dynamic, morphological, and contextual features of cells within a culture.

Recent studies have adopted representation learning techniques to capture cellular dynamics directly from raw data. Several self-supervised approaches leverage autoencoder-based architectures to learn latent representations of cell morphology and dynamics, which are subsequently analyzed in search for their biological relevance. For example [9], learned representations from time-lapse sequences and related principal components of the latent space to handcrafted morphological features, enabling post-hoc interpretation. Similarly [10], employed vector-quantized autoencoders to learn discrete representations for cell-cycle classification. While these methods provide useful representations of cellular dynamics, they primarily focus on cell-cycle stage rather than direct prediction of divergent outcomes of cells under treatment.

[11] explored cell fate prediction in multicellular contexts. The study proposed an interpretable graph neural network framework to model cell fate decisions in tissues, explicitly incorporating cell–cell interactions through graph structures derived from live imaging data. Their work highlights the importance of spatial context and collective dynamics but does not consider intracellular signaling dynamics in the prediction.

[12,13] introduced autoencoder-based cell representations to predict cell fate. Latent representations were analyzed for their link to the morphological and intensity-based features and predictability.

Among these studies, a systematic investigation of when predictive information emerges over time and how temporally localized cues relate to interpretable cellular features remains limited. In particular, none of the studies explored the predictive power of Transformer models and their interpretability via the attention mechanism. Transformers represent powerful architectures to process sequential data, such as time-lapse recordings. Their use in the study of cellular dynamics, particularly in the context of chemotherapeutic treatment and dynamic molecular reporters, has not been demonstrated yet.

Here, we develop a deep learning model to predict the fate of individual cancer cells following exposure to chemotherapeutic treatment. We propose a Transformer-based architecture that operates on temporal sequences of center-cropped image patches extracted from long-term time-lapse microscopy data. The input captures not only cellular morphology but also the dynamic activity of multiple transiently active molecular markers, including p53, circadian, and cell-cycle reporters. Importantly, the model predicts whether a cell undergoes apoptosis or mitosis directly from raw image sequences, without relying on predefined morphological features, allowing it to learn task-relevant representations in a data-driven manner.

We investigate not only the extent to which cell fate can be predicted from video-encoded cellular trajectories but also develop model explainability strategies to address two key questions: (1) when during a cell’s lifetime fate decisions become predictable, and (2) which signals are most informative for those decisions. Our work demonstrates that the combination of rich time-lapse microscopy data, temporal modeling, and attention-based analysis enables the extraction of meaningful biological signals and facilitates the discovery of determinants of cell fate following chemotherapeutic treatment.

The contributions of this work can be summarized as follows:

- We introduce a Transformer-based model for predicting cell fate from long-term single-cell raw image sequences.
- We demonstrate prediction accuracy on a challenging, imbalanced live-cell imaging dataset and show that prediction of cell fate is indeed possible from videos of cell trajectories.
- We perform temporal truncation experiments to assess how predictive information is distributed across time.
- We propose an attention-based explainability framework that links temporally important frames to interpretable morphological and intensity-based features.

## 2 Methods

### 2.1 Dataset

We use a publicly available live-cell imaging dataset [14], comprising 274 time-lapse fluorescence microscopy videos. Each video consists of 186 frames with a spatial size of 1024×1024 pixels, acquired every 30 minutes over a continuous period of 93 hours.

Out of the 274 videos, 118 capture cell cultures under treatment with the chemotherapeutic agent cisplatin. Cisplatin induces DNA damage and was administered 48 hours after the start of imaging at three different dosage levels (high, medium, and low). Three fluorescent reporters were used to monitor key cellular pathways: CFP-hGeminin, which reports on cell-cycle progression by indicating S/G2/M phases; NR1D1::VNP, a fluorescent circadian clock and metabolism reporter reflecting cellular stress and circadian rhythm activity; and a p53 transcriptional activity reporter, capturing stress- and DNA-damage–related signaling dynamics. Together, these channels provide a rich temporal record of molecular signaling and cellular state transitions.

In each video, cells are tracked and assigned unique cell identifiers. All annotations were performed manually. Each cell was tracked by marking its centroid across consecutive frames, with mitotic events recorded at the frame in which a parent cell separated into two daughter cells and apoptotic events recorded at the first frame exhibiting clear signs of cell death, such as morphological collapse. In total, the dataset contains approximately 51,000 individual cell trajectories (averaging ~187 cells per video), including about 18,000 mitotic events and 3,500 apoptotic events. The remaining sequences do not carry a cell fate label, either because the cell left the field of view before a fate could be determined or because the recording ended before the fate occurred.

To predict fate of individual cells, we extracted temporal image sequences by center-cropping each annotated cell at a spatial resolution of 112×112 pixels around its centroid, ensuring that the entire cell body was captured throughout the sequence. Each sequence spans from the first appearance of the cell up to the frame preceding the cell event. If *t* denotes the event frame, i.e., *t*he first frame showing clear apoptotic collapse, or the first frame in which two separate daughter cells are visible, we include only frames 0 to t−1 as model input. Thereby, the visible collapse or completed division are never used in prediction. Each sequence was paired with its corresponding cell fate label - mitotic vs. apoptotic outcome. For our approach, we exclusively included cells with annotated mitotic or apoptotic fates. Furthermore, we included only sequences longer than 10 frames. When a crop extended beyond the image boundaries, zero-padding was applied to preserve a fixed patch size.

The sequence-length distributions differ across both fate and dose (S1 Fig). Apoptotic sequences are considerably longer than mitotic ones (median 73 vs. 45 frames; mean 74.0 vs. 44.2, S1 Figa). Control and cisplatin-treated cells have a similar median sequence length (57 vs. 58 frames, S1 Figc), but the treated cells show a broader distribution with a longer tail toward high sequence lengths (mean 64.4 vs. 56.7, S1 Figb). Across the three cisplatin doses the length distributions are broadly comparable, with a slight decrease at lower doses (median 65, 57, and 51 frames for high, medium, and low dose, respectively).

The composition of the dataset with respect to fate and dose in summarized in Table 1. The proportion of apoptotic events increases with dose, rising from 0.9% of events in the control condition to 27.4% at the highest dose, while the proportion of mitotic events decreases correspondingly. Since the control group contains only 41 apoptotic sequences, precludes a robust characterization of apoptotic dynamics under control condition. Our interpretability analyses therefore focus on the cisplatin-treated sequences. A large subset of sequences originates from videos for which the information on the administered dose is not available (denoted as “Unknown”). These cells are excluded from any dose-stratified or treatment-specific analysis.

**Table 1:** Composition of the dataset by cell fate and cisplatin dose.

Fate	Control	Low	Medium	High	Unknown	Total
Apoptosis	41	411	570	1,219	2,215	4,456
Mitosis	652	960	761	801	14,268	17,442
**Total**	693	1,371	1,331	2,020	16,483	21,898

### 2.2 Classification

An overview of the proposed Transformer-based cell fate prediction framework and the attention-guided analysis pipeline is shown in Fig 1.

**Fig 1:**
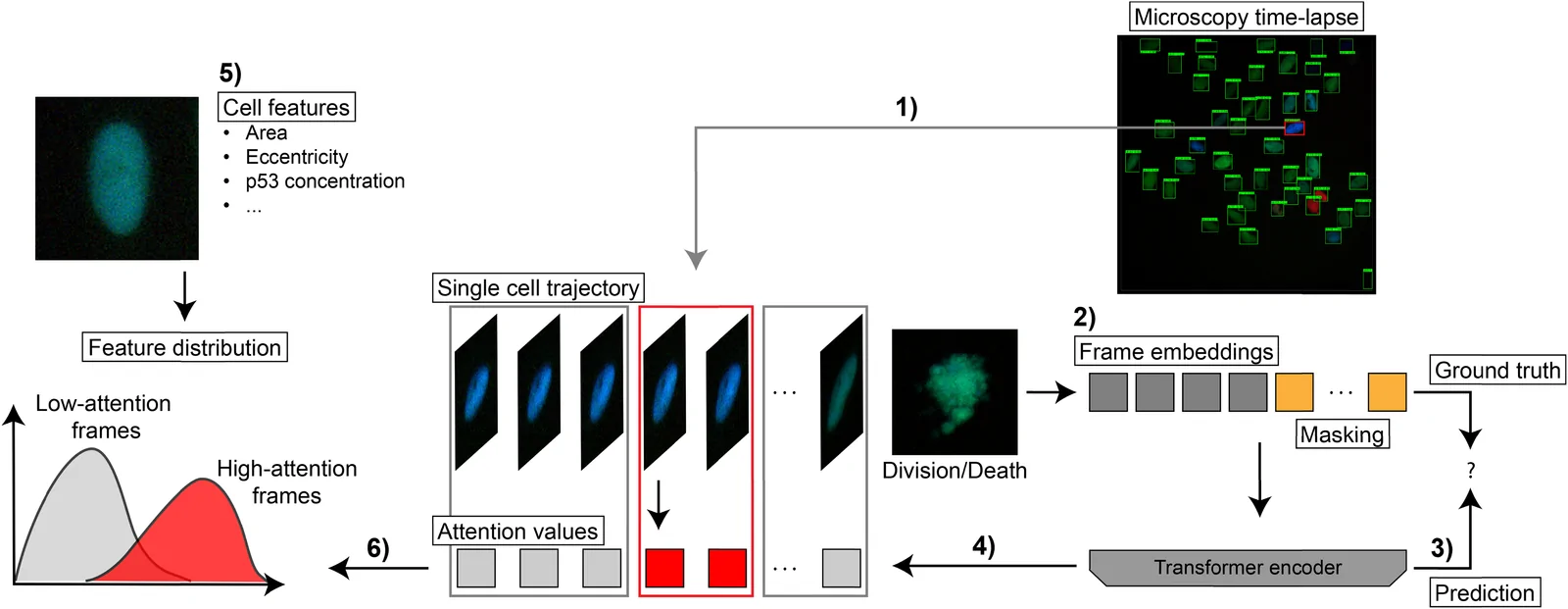
Overview of the proposed cell fate prediction framework and the explainability using temporal attention analysis. **1)** From time-lapse microscopy recordings, we extract single-cell trajectories. Each trajectory starts at the first appearance of a cell and ends at the last frame prior to the fate event (division or death). **2)** For each cell patch, frame-level embeddings are computed using a ResNet-50 backbone. We randomly mask a portion of frames at the end of each trajectory during training. **3)** The sequence of frame embeddings is processed by a Transformer encoder, which integrates temporal information to predict the final cell fate. **4)** To enable interpretability, we extract the attention weights assigned to individual frames and categorize them into high-attention and low-attention frames. **5)** In parallel, we compute handcrafted cell features for each patch (e.g., area, eccentricity, p53 concentration). **6)** Finally, we compare the feature distributions of high- and low-attention frames to identify biologically meaningful patterns associated with the model’s decision-making process.

Each input consists of a time-lapse sequence of cropped single-cell images. Individual cell images are first processed independently by a ResNet50 backbone to extract frame-wise (2048-dimensional) feature embeddings. These embeddings represent input tokens to our Transformer model. [cls] token is prepended to the sequence of image tokens to serve as a global representation of the entire trajectory.

To encode temporal order, we add sinusoidal positional embeddings [15] to all elements of the sequence, computed on absolute frame indices starting at the first appearance of the cell. The resulting representation is then input into a Transformer encoder composed of 8 identical layers, each employing multi-head self-attention with 4 attention heads. This encoder models long-range temporal dependencies and interactions between time points across the full cell trajectory.

On top of the encoder output, we apply an additional attention layer to aggregate temporal information and capture the relative relevance of individual frames for the final prediction. Layer normalization is applied both before and after this attention layer to stabilize optimization and improve representational consistency.

Finally, the transformed [cls] token is passed through a linear classification head to predict the cell fate based on the full temporal sequence.

#### 2.2.1 Morphology-feature baseline.

As a baseline that isolates the contribution of the learned image embeddings, we consider a variant in which the ResNet-50 backbone is replaced by a segmentation-based feature extractor, while the rest of the architecture is left unchanged. For each frame, cells are segmented with Cellpose [16] and the same morphological features are extracted as described in Sect 2.3. The size-related features are log-transformed and all features are standardized using training-set statistics. The resulting per-frame feature vectors are embedded by a small MLP and passed to the same Transformer encoder in place of the ResNet-50 embeddings. The only change to the architecture is the Transformer’s input embedding dimension, set to match the feature-embedding output rather than the ResNet-50 output. All other components and training settings are kept identical, so that the comparison isolates the effect of the representation.

### 2.3 Explainability

In addition to predicting cell fate, we investigate which frames within a temporal sequence are the most influential for the model’s decision-making process and how these frames differ from the less informative ones. To this end, we leverage the attention mechanism of the model as a proxy for temporal importance.

During inference, we extract attention weights from the final attention layer of the network. Given a sequence *s* of length *N*, we denote the attention weights assigned to individual frames $\{f_{s,i}{\}}_{i=1}^{N}$ by $\{a_{s,i}{\}}_{i=1}^{N}$. To identify the most relevant frames within a sequence, we define a sequence-specific threshold $\tau_{s}$ as the 90th percentile of the attention weights of *s*. Using this threshold, we partition the set of frames into two disjoint subsets:


$$\begin{array}{c} {ℋ}_{s}:=\{f_{s,i}|a_{s,i}\geq \tau_{s}\} \end{array}$$ (1)

$$\begin{array}{c} {ℒ}_{s}:=\{f_{s,i}|a_{s,i}<\tau_{s}\} \end{array}$$ (2)

Thus, ${ℋ}_{s}$ contains the 10% of frames within sequence *s* receiving the highest attention weights, while ${ℒ}_{s}$ comprises the remaining frames.

Before analyzing the content of the high-attention frames, we first verify that these frames are indeed the most influential for the model’s prediction. To this end, we perform a masking experiment on the test set that measures the impact of masked frames on the prediction. For each sequence of length *N*, we rank all frames by their attention weight and mask a fraction ρ∈{0.1,0.2,...,0.9} of the sequence, corresponding to $n_{\rho }=round(\rho N)$ frames. We compare three selection strategies each removing $n_{\rho }$ frames out of the: (i) the highest-attention frames, (ii) the lowest-attention frames, and (iii) randomly chosen frames. We emphasize that the masked frames are selected directly from the ranked attention weights of the entire sequence at each fraction ρ, independently of the fixed high-/low-attention partition defined above.

To search for distinguishing features between high- and low-attention frames, we quantify morphological and intensity-based characteristics of cells in all video frames. Individual cells are segmented using Cellpose [16], and visual features are computed from the resulting segmentation masks using *scikit-image* [17]. To avoid confounding effects from neighboring cells appearing in the cropped patches, only the centrally located cell is considered for feature computation.

The extracted features include cell area, perimeter, equivalent diameter, eccentricity, solidity, circularity, and the mean intensity of each fluorescence channel.

For each sequence and feature, we conduct permutation tests with 50,000 iterations to assess whether feature distributions differed significantly between ${ℋ}_{s}$ and ${ℒ}_{s}$. This yields one p-value per feature and sequence. In addition to statistical significance, we quantify the magnitude and direction of differences using Cliff’s delta [18]. Unlike Cohen’s *d*, Cliff’s delta does not assume normality or equal variances and is robust to skewed distributions and unequal sample sizes [19].

Each sequence therefore contributes a single p-value and a single Cliff’s delta per feature. Aggregating over all *n* analyzed sequences (where *n* denotes the number of sequences in the respective dataset) thus yields *n* p-values and *n* Cliff’s delta values per feature. To assess global consistency of within-sequence effects, we additionally compute the median Cliff’s delta and its 95% confidence interval. A median effect size whose confidence interval is shifted away from zero is interpreted as evidence of a consistent effect, with the magnitude indicating the strength and direction of the association.

### 2.4 Training

We randomly split our cell trajectories into training, validation, and test sets using an 0.8/0.1/0.1 ratio, while approximately preserving the distribution of mitotic and apoptotic events across all splits. During training, we used a batch size of 4 and to address the pronounced class imbalance, we employed a weighted random sampler to approximately balance mitotic and apoptotic samples. As cell trajectories vary in temporal length, sequences were padded at the end of the sequence with empty tokens to match the maximum sequence length within each batch. During training, padded positions were masked within the Transformer encoder to ensure that they do not contribute to the attention computation. At evaluation time, sequences are processed individually and no padding is applied.

To improve generalization, we applied a range of data augmentations independently and stochastically during training. The augmentation pipeline includes random geometric transformations, such as horizontal and vertical flips, as well as appearance-based augmentations including color jittering and Gaussian blurring. In addition, we applied a sequence-level augmentation by randomly masking a contiguous segment at the end of each sequence, with the masked portion ranging between 10% and 50% of the total sequence length. Each augmentation is applied with a fixed probability, increasing the diversity of the training data and improving model robustness.

The ResNet backbone, Transformer encoder, final attention layer, and classification head were trained from scratch using binary cross-entropy loss. Optimization was performed using the Adam optimizer with a learning rate of $1\times 10^{-5}$ and a weight decay of $1\times 10^{-3}$. Training was conducted for up to 86 epochs with early stopping based on the validation loss, and the model parameters corresponding to the best validation performance were retained. All experiments were conducted on a system equipped with an AMD EPYC 7662 CPU, 150 GB of RAM, and an NVIDIA Tesla A100-SXM4 GPU with 40 GB of memory. Reproducibility was ensured through fully deterministic training and evaluation. All experiments were run with fixed random seeds across Python, NumPy, and PyTorch, and deterministic computation settings were enforced to guarantee identical results across runs.

## 3 Experiments and results

In this section, we present experimental results evaluating both the predictive performance and explainability of our model for single-cell fate prediction. We first report classification performance, compare our model against a morphology-based baseline, and extend it to a three-class prediction. We then use temporal truncation experiments to characterize how predictive information is distributed across time, and attention-based explainability to identify the temporal signals and cell characteristics that drive the model’s predictions

### 3.1 Classification

Classification performance was evaluated on a held-out test set of cell trajectories. The test set comprised 2,089 sequences, including 439 apoptotic and 1,650 mitotic events, reflecting a strong class imbalance of the entire dataset.

Our model achieved a balanced accuracy (BAcc) of 0.94 and an F1-score of 0.9326 on the test set. As shown in the confusion matrix in Fig 2a, the proposed approach correctly identified 91.3% of apoptotic samples (401 out of 439), demonstrating a high recall for the minority class. Only 8.7% of apoptotic instances were misclassified as mitotic events.

**Fig 2:**
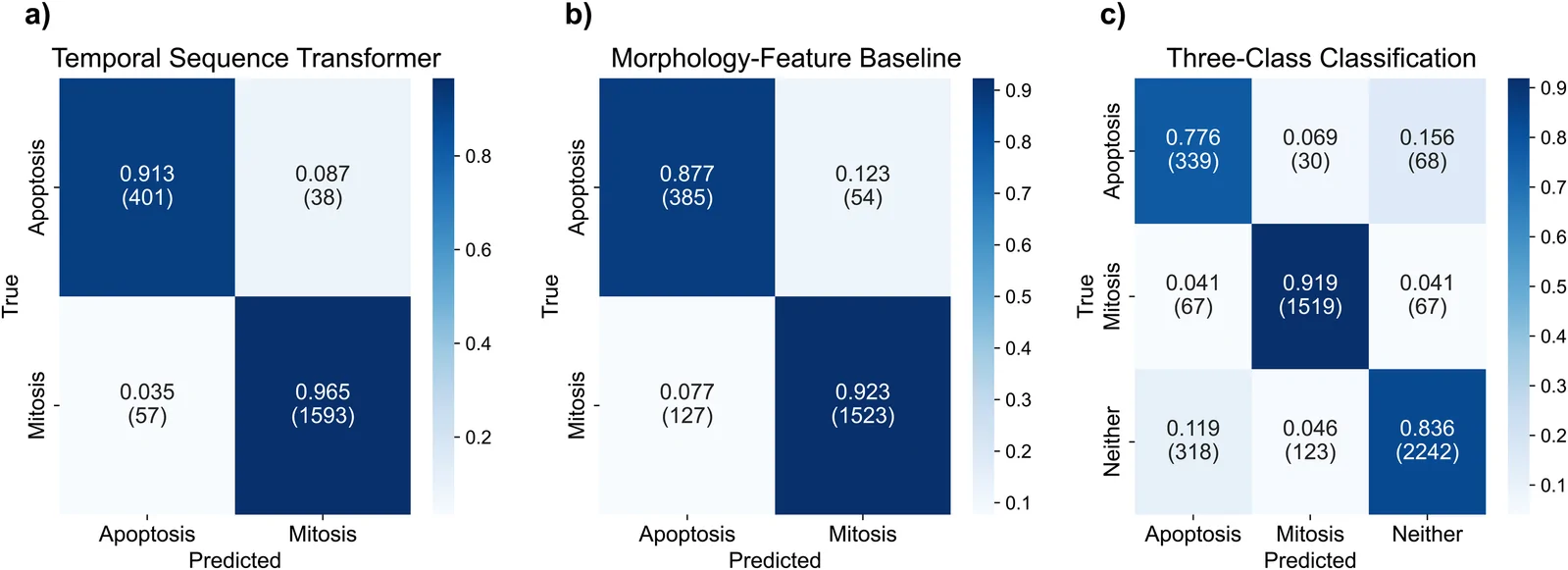
Classification evaluation. Confusion matrices normalized over the ground-truth labels, with absolute counts shown in parentheses. **a)** Full model, our temporal sequence Transformer operating on raw image sequences. **b)** Morphology-feature baseline, in which the ResNet-50 backbone is replaced by handcrafted segmentation-based features while the Transformer encoder is left unchanged. **c)** Three-class setting, distinguishing apoptotic, mitotic, and “neither” cells.

At the same time, the model maintained a low false-positive rate for apoptosis prediction, with only 3.5% of mitotic samples incorrectly predicted as apoptotic. This indicates that the high recall for the minority class was not achieved at the expense of excessive number of false positives. Overall, these results demonstrate that the proposed model reliably discriminates apoptotic from mitotic events despite the pronounced class imbalance in the dataset.

**Morphology-feature baseline.** To assess the information content of the learned CNN embeddings, we compared our model against a baseline operating on handcrafted morphological features only (Sect 2.2.1). This baseline achieved a BAcc of 0.90 and an F1-score of 0.877 (Fig 2b), below the 0.94 BAcc and 0.9326 F1-score of the full model operating on raw image sequences. The handcrafted morphological features alone allow the model to reach strong predictive performance. However, image embeddings increase this performance further.

**Three-class classifier.** For many of cell in our data the fate is undetermined - these cells do not divide or die within the timespan of our recordings or leave the field of view. We next trained the model to distinguish three classes of sequences - mitotic, apoptotic, and “neither”. Cells labeled as “neither” have not yet undergone a fate transition, their eventual fate is unobserved rather than absent. Therefore, “neither” class inevitably overlaps with the two cell fate classes in feature space. With this caveat, the model achieved a balanced accuracy of 0.8434 and a macro F1-score of 0.7946 (Fig 2c). The most errors stem from the confusion between the “neither” and the apoptosis class. We therefore regard three-class classification as feasible but inherently limited by the ambiguity of the “neither” label, and retain the binary fate prediction as the focus of this work.

#### 3.1.1 Temporal truncation.

Since a cell might undergo visual changes shortly before its death or division that clearly reveal its fate, we next performed an experiment in which we removed an increasing number of frames at the end of each sequence and inspected the accuracy of prediction on this way shortened cell trajectories. We evaluated the model on the corresponding test set while progressively truncating the final 0,5,10,...,50 frames of each sequence. Sequences shorter than the respective truncation length were excluded from this analysis. The resulting performance is shown in Fig 3a.

**Fig 3:**
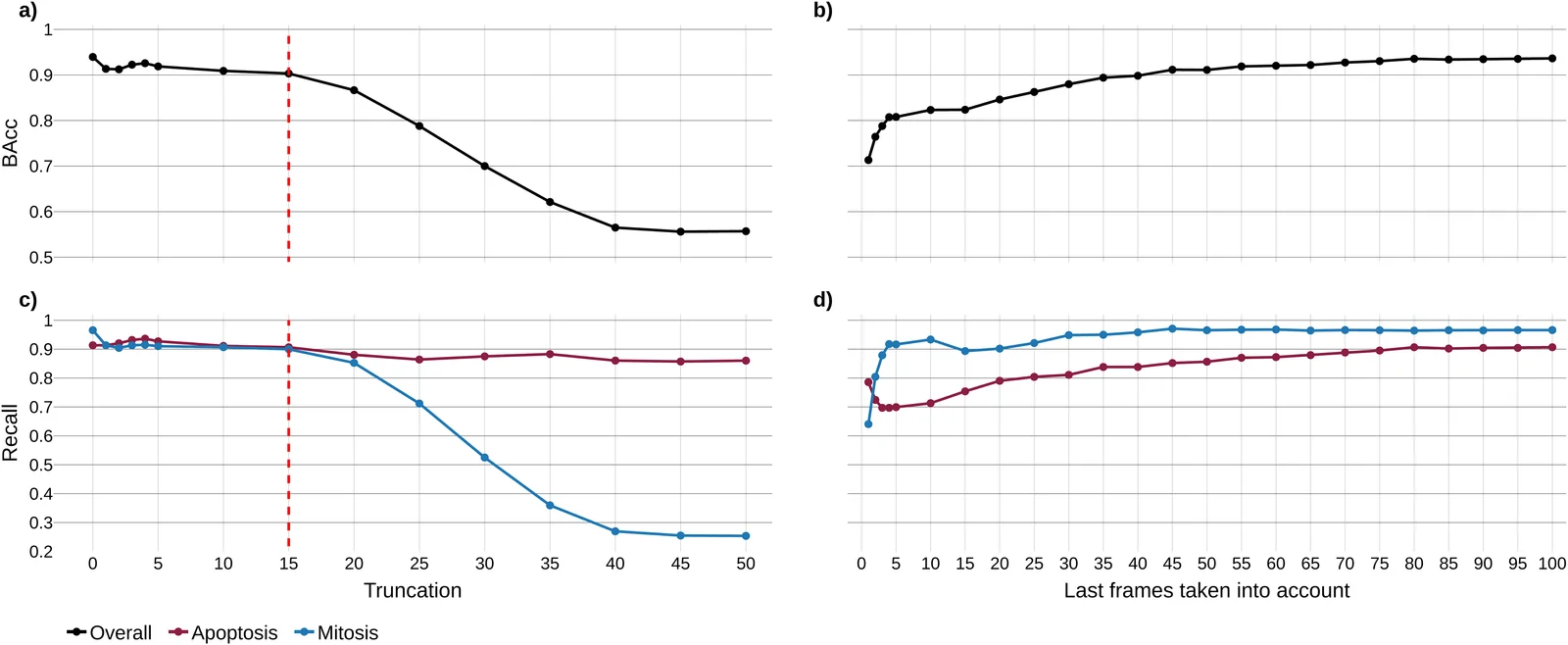
Temporal truncation analysis to assess the importance of late parts of the cell trajectory for its fate prediction. **a)** Balanced accuracy when progressively truncating sequences by removing an increasing number of frames from the end of each trajectory. **b)** Balanced accuracy when restricting the model input to only the last k frames of each sequence. **c)** Class-wise recall under progressive truncation from the sequence end, reported separately for apoptosis and mitosis. **d)** Class-wise recall when predictions are based solely on the last *k* frames, highlighting the contribution of late temporal information for each cell fate.

When evaluating our model on the whole test set, we again observed a BAcc of 0.94. Omitting the final 5 frames resulted in a moderate performance decrease to a BAcc of 0.919. Performance remained relatively stable when up to 15 frames were omitted (BAcc: 0.903). In contrast, truncating 20 or more frames led to a pronounced decline in performance, which continued steadily with increasing truncation length, reaching a BAcc of 0.565 when 40 frames were removed. Omitting up to 50 did not further degrade the performance by a large amount. Class-specific recall values (Fig 3c) further reveal distinct truncation sensitivities for the two cell fate outcomes. Recall of apoptotic sequences remained comparatively stable, decreasing by only 0.053 from 0.913 without truncation to 0.860 when the final 50 frames were removed, and staying above 0.9 up to a truncation of 15 frames. In contrast, recall of mitotic sequences was far more sensitive to the removal of final frames. Removing only the single last frame already reduced mitotic recall by 0.052 (from 0.965 to 0.913), removing 5 frames reduced the recall further to 0.910. Mitotic recall then remained relatively stable until a truncation of approximately 15 frames (recall:0.900), followed by a substantial decline to 0.254 when the final 50 frames were removed. These results indicate that the temporal distribution of predictive signal differs between the two cell fate classes. In particular, mitotic sequences appear to be predictable from signals late in the sequence, with the final ~8 hours prior to the event contributing most strongly to discriminative performance, and earlier time points providing only limited predictive information. In contrast, apoptotic sequences which are predictable from signals across a broader temporal window.

As a complementary perspective on the same question, we inspected the change of predicted class probability with the increasing sequence length. A dramatic change of this probability value would indicate existence of “trigger events” that determine cell fate happening later in the sequence. For each cell, we predicted cell fate based on sequences of increasing length from the first frame of the sequence up to frame $t^{\prime }$ with $t^{\prime }$ increasing towards the cell event. Trigger events were identified as the last crossing of the probability curve of the 0.5 threshold. Representative mitotic and apoptotic sequences with their trigger events are shown in Fig 4.

**Fig 4:**
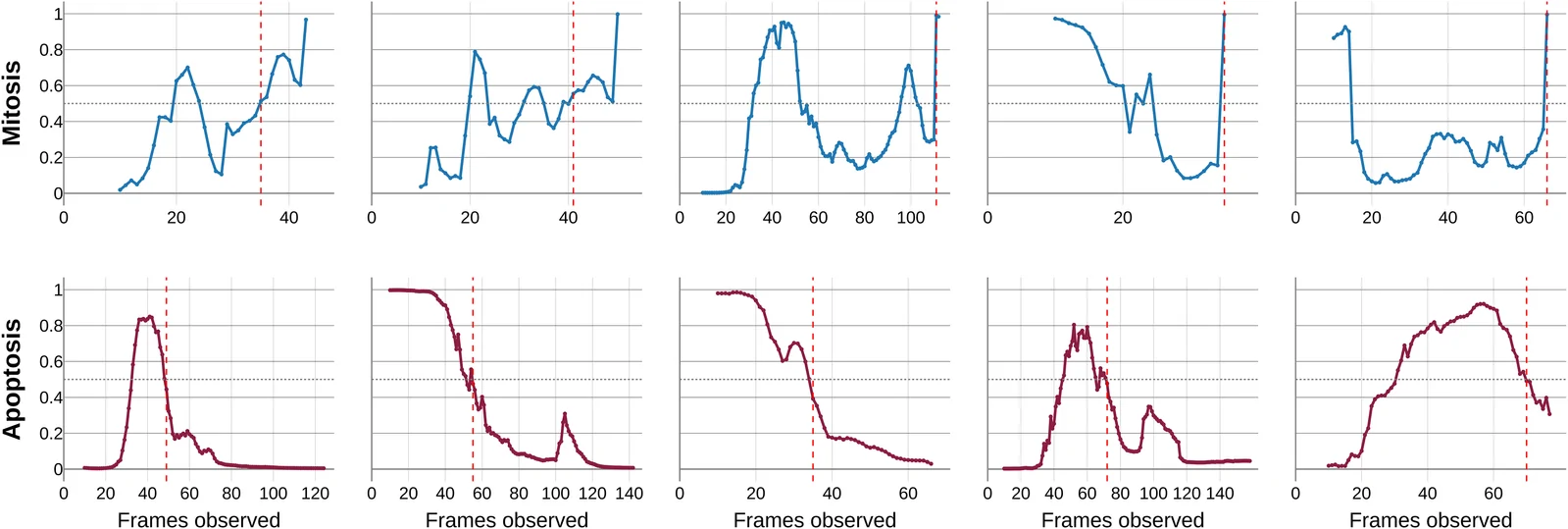
Causal prediction trajectories and trigger events. Representative per-sequence prediction trajectories for mitotic (top row) and apoptotic (bottom row) cells. For each cell, the model’s predicted probability is shown as a function of the sequence length, from the start of the sequence up to ***t*.** The dashed line marks the 0.5 decision boundary, and the identified trigger event, defined as the last crossing of this boundary, is highlighted.

This analysis was restricted to the 1,994 sequences of the test set that were correctly classified based on the full sequence. Of the 1,593 mitotic sequences we analyzed, 1,312 (~80%) exhibited a trigger event with crossings most frequently occurring 20–35 frames prior to division (see S2 Fig) corresponding roughly to the beginning of the S-phase. Of the 401 apoptotic sequences, only 212 (~48%) exhibited a trigger event, and these crossings were distributed across the entire observed window without a prevalent temporal position. The remaining sequences were assigned to the correct class from the earliest evaluated prefix onward and therefore did not cross the decision boundary.

Because apoptotic sequences tend to be longer than mitotic ones (see S1 Fig), there are more apoptotic than mitotic sequences that are analyzed in every step of the truncation test. To verify that the predictability of apoptosis is not an artifact of this length asymmetry, we repeated the truncation analysis on a length-matched subset of the test set, in which apoptotic and mitotic sequences were sampled to share the same length distribution and all retained sequences were at least 50 frames long. On this matched subset (S3 Fig) apoptosis recall remained high across the full truncation range, decreasing only marginally from 0.91 at no truncation to 0.81 when removing the final 50 frames, whereas mitosis recall declined more steeply. This confirms that the model identifies information predictive of apoptosis well before the death event even when sequence length is controlled for, ruling out the length distribution as an explanation for the effect.

In a further analysis, we examined how many frames at the end of a sequence were sufficient to predict the cell fate. Instead of truncating the end of the sequence, we retained only the last *k* frames and discarded all preceding frames. Fig 3b shows the corresponding results. Using only the last *k* = 1 frame yielded a BAcc of 0.713, which rose steadily as further frames were added, to 0.808 at *k* = 5. Performance continued to increase more gradually with additional frames, reaching near-optimal performance at approximately *k* = 80 frames (BAcc: 0.935), after which additional frames provided only marginal improvements.

Here, we also analyzed class-specific performance by computing recall for each event type while restricting the model to only the last *k* frames (Fig 3d). Mitotic sequences could be identified from very few frames. Using the last *k* = 1 frames yielded a recall of 0.641, which rose steeply as the next few frames were added, to 0.804 at *k* = 2, 0.879 at *k* = 3, and 0.918 at *k* = 4. Recall then remained high across a broad range of *k*, stabilizing above 0.96 from approximately *k* = 45 onward. This shows only the last few frames suffice for the model to predict mitotic events. In contrast, apoptotic sequences required substantially more temporal context for reliable prediction. When restricting the sequences to the last *k* = 5 frames, recall was only 0.699, and it increased progressively as additional frames were included, reaching 0.907 at *k* = 80 frames, after which further increases in sequence length did not yield additional performance gains. Again, this suggests that signals predictive of cell division appear shortly prior to the event while signals predictive of cell death can be distributed over longer time spans.

### 3.2 Explainability

Beyond predictive performance, understanding how and when the model arrives at its decisions is critical for model interpretation. Unless stated otherwise, the following analyses are restricted to sequences originating from videos in which cisplatin was administered. This lets us specifically study the temporal cues predictive of divergent cell fate decisions under chemotherapeutic treatment. We first verify that the attention weights faithfully reflect the frames the model relies on for its predictions. We then use the attention mechanism to investigate (1) when during a cell’s trajectory predictive information appears and (2) which cell features contribute most to fate discrimination. Finally, we examine the full temporal trajectories of attention and of the interpretable features.

#### 3.2.1 Attention validation.

A prerequisite for interpreting attention weights as a measure of temporal importance is that the high-attention frames are indeed the frames the model relies on for its prediction. To confirm this, we progressively masked an increasing fraction of frames in a sequence that are either the highest-attention frames, the lowest-attention frames, or are randomly selected. The results are shown in Fig 5.

**Fig 5:**
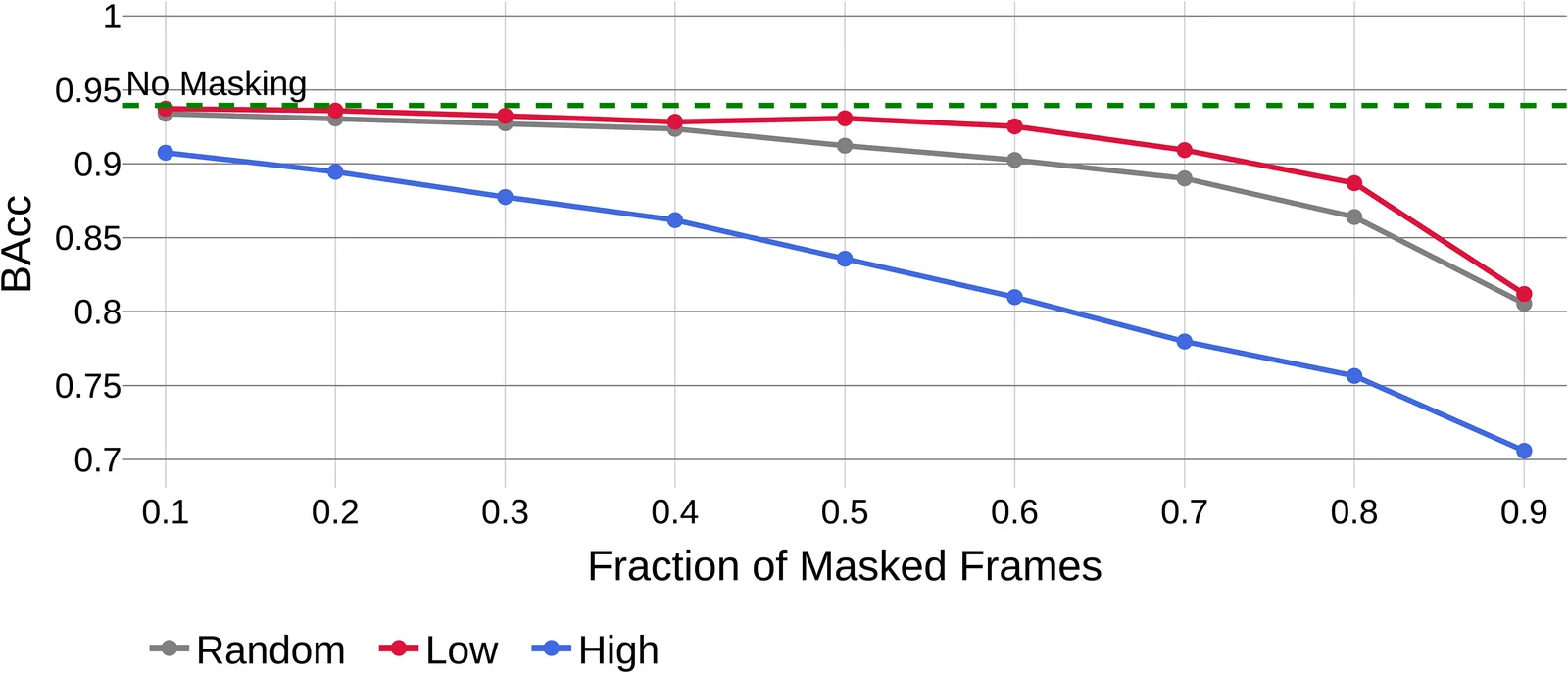
Attention Validation. Balanced accuracy as a function of the masked frames, for three masking strategies: highest-attention, lowest-attention, and random frames.

Across all masking fractions, removing the highest-attention frames degraded performance the most, more than removing random or low-attention frames. At a masking fraction of 0.1, BAcc dropped from 0.939 to 0.907 when masking high-attention frames, compared to only 0.934 and 0.937 for random and low-attention masking, respectively. This gap widened as more frames were removed. At a fraction of 0.5, masking high-attention frames reduced BAcc to 0.836, whereas random and low-attention masking retained 0.912 and 0.931. Notably, masking low-attention frames consistently degraded performance the least, even less than random masking, indicating that the frames receiving the lowest attention were also the least informative for the prediction. Together, these result confirm that the attention weights are faithful to the model’s decision process. Frames identified as important based on the attention mechanism are those whose removal most strongly impairs prediction, justifying their use as a basis for the subsequent feature analysis.

#### 3.2.2 Attention focus.

To corroborate the findings based on sequence truncation and to identify what are the signals predictive of particular cell fate, we next performed attention-based explainability analysis. Following the procedure described in Sect 2.3, we identified the high-attention and low-attention frames ${ℋ}_{s}$ and ${ℒ}_{s}$ in all sequences s∈S.

In Fig 6, we aggregated attention weights across all sequences by aligning them to the final frame of each sequence. Specifically, we considered up to 50 frames preceding the cell event and computed the mean attention weight per frame. Attention values were normalized using the global minimum and maximum attention weights. Only sequences that have been correctly classified by the model were included in this analysis.

**Fig 6:**
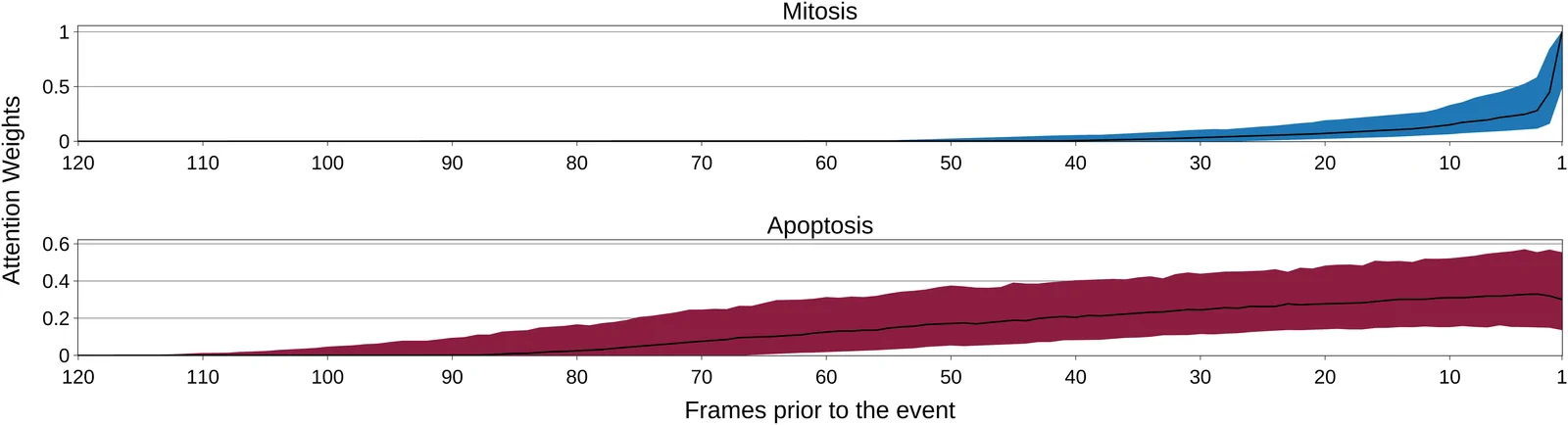
Attention patterns. Aggregated attention weights across correctly classified sequences, aligned to the final frame and normalized by the global minimum and maximum attention values.

In sequences of dividing cells, the aggregated attention profile shows a pronounced peak at the final frame, indicating that the model’s predictions relied predominantly on information from the frames shortly prior to the event. The average normalized attention weight was markedly lower in the earlier frames, at 0.62 in the second-to-last frame and 0.22 at the 10th frame before the event. This suggests that, for mitosis, the most discriminative information was concentrated in the final frames of each trajectory.

In contrast, apoptotic sequences exhibited a broader and more gradual temporal distribution of attention. Notably, the maximum average attention was observed not at the final frame, but at the 5th frame preceding the event. Elevated attention weights persisted across a substantial temporal range, with a gradual decline towards earlier frames. The final frame before the event retained a relatively high normalized attention weight of 0.88, while 30 frames before the event still exhibited an average attention weight of approximately 0.42. Attention weights decreased more markedly only beyond this range, reaching 0.07 at the 45th frame prior to the event. These findings indicate that, for apoptotic sequences, informative signals were distributed across a wider temporal context rather than being confined to the immediate vicinity of the final event.

#### 3.2.3 Predictive features.

Having identified frames important for prediction, we next investigated which morphological and intensity-based features distinguish high-attention frames from less informative ones. Feature quantification and statistical testing followed the procedure outlined in Sect 2.3. Not all cells were correctly segmented. The frames with missing segmentation were discarded from the feature analysis.

We used permutation test and Cliff’s delta to identify significantly different features between the two groups of frames. We repeated this for all sequences and calculated the median Cliff’s delta and its 95% confidence interval to determine the direction of the feature change. The results are shown in Fig 7.

**Fig 7:**
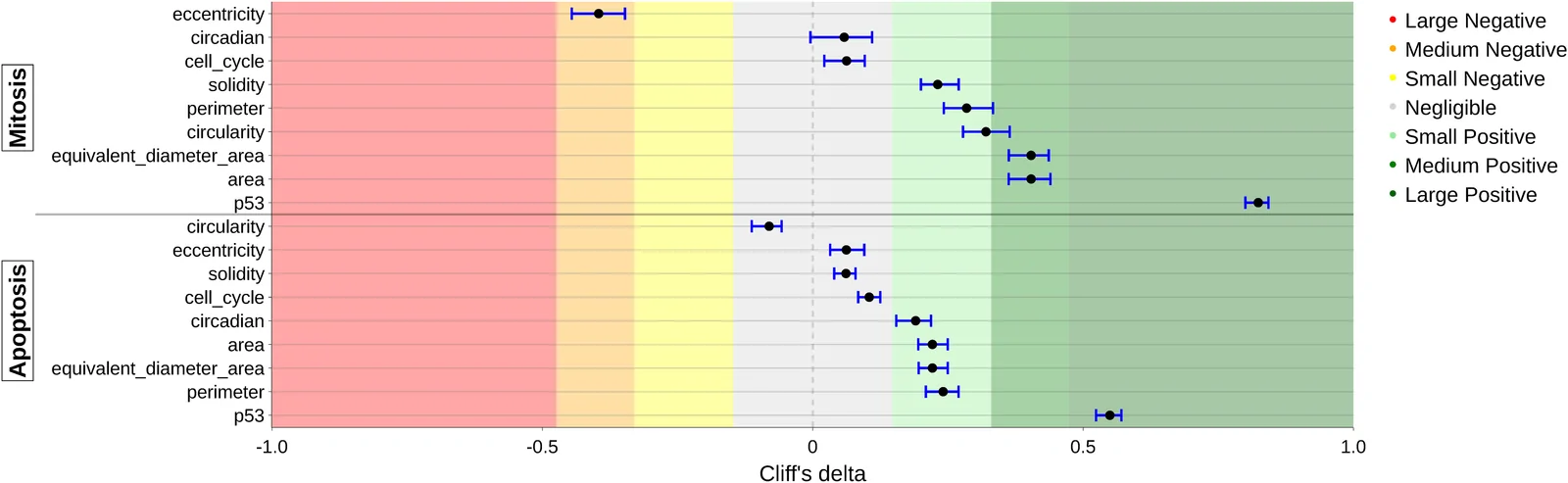
Effect size analysis. For each feature, the median Cliff’s delta and its 95% confidence interval are shown separately for mitosis and apoptosis. Positive values indicate higher feature values in high-attention frames compared to low-attention frames, whereas negative values indicate the opposite. The background shading denotes commonly used effect size categories (negligible, small, medium, and large effects), facilitating interpretation of the magnitude and direction of the observed differences.

For mitotic sequences, several features exhibited consistent differences between high- and low-attention frames. Most notably, the p53 channel showed a pronounced positive effect, indicating higher p53 intensity in frames that received high attention. Area-related features, including cell area and equivalent diameter, exhibited medium positive effects, while perimeter showed a smaller positive effect. Circularity displayed a small to medium positive effect, solidity a small positive effect, and eccentricity a medium negative effect. This pattern suggests that frames predictive of division were characterized by cells that appear larger and more rounded. Fig 8 shows a mitotic sequence where a progressive increase in cell size followed by pronounced mitotic rounding toward the end can be observed.

**Fig 8:**
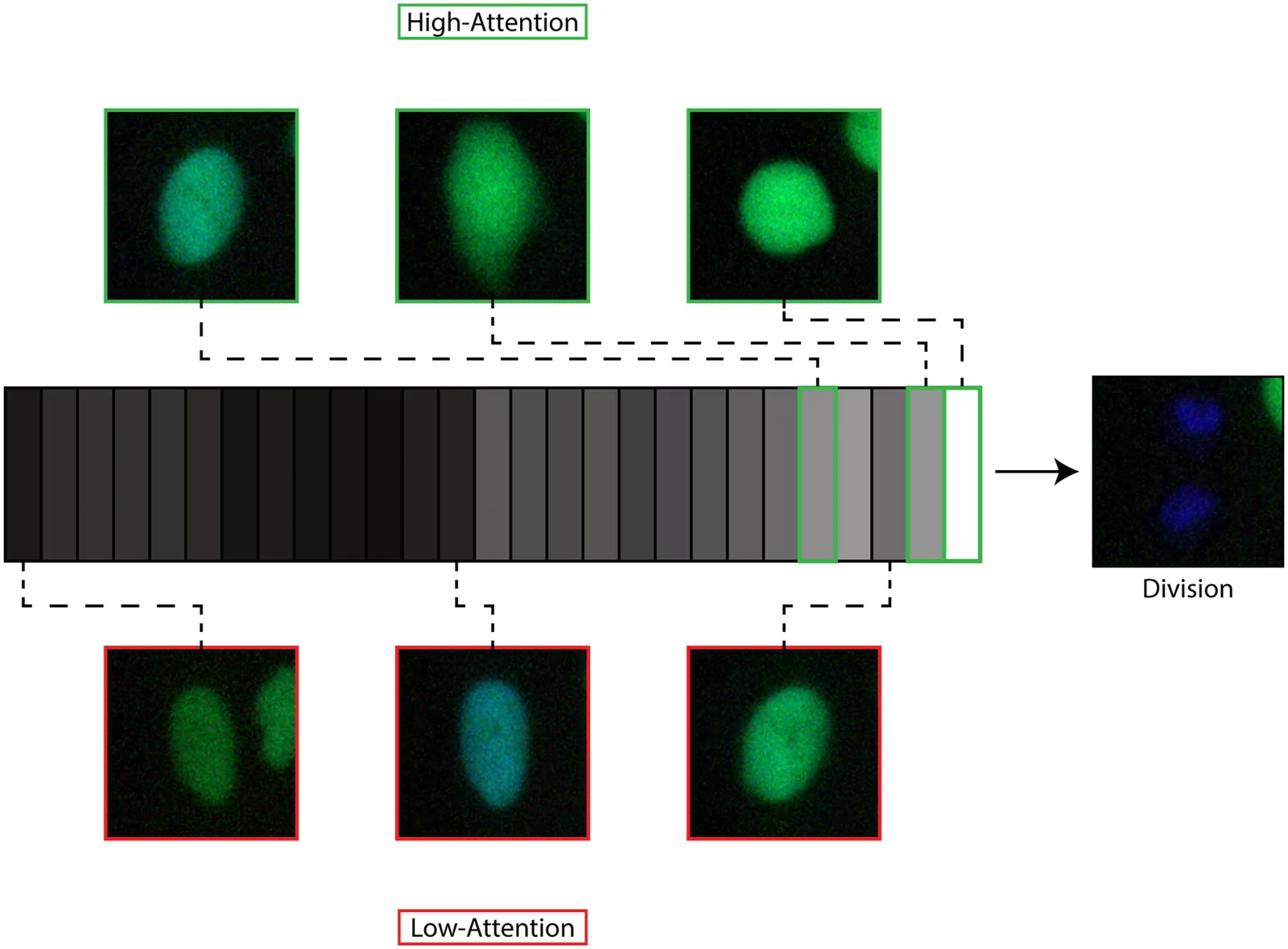
Example mitotic sequence. Illustrative mitotic trajectory with corresponding temporal attention weights. The sequence depicts progressive cell growth followed by characteristic mitotic rounding in the frames immediately preceding division. Frames assigned high attention by the Transformer model are highlighted in green, whereas representative low-attention frames are marked in red. Attention weights were normalized across the sequence.

For apoptotic sequences, small positive effects were observed for equivalent diameter area, cell area, perimeter, and circadian features, indicating modest but consistent differences between high- and low-attention frames across these properties. In contrast to mitosis, no pronounced difference between high- and low-attention frames were observed for circularity and eccentricity in apoptotic sequences. This suggests that while high-attention frames in apoptotic sequences were also characterized by larger cells, they were not necessarily round, but instead retain their original shape. Notably, p53 again exhibited a large positive Cliff’s delta median, highlighting elevated p53 intensity as a dominant characteristic of frames important for apoptosis prediction. Interestingly, high-attention frames were also characterized by a modest increase in the intensity of the circadian reporter channel. This is in line with previous studies establishing a relationship between circadian signals and apoptosis at the single-cell level [20,21]. An example of an apoptotic sequence is shown in S8 Fig.

Finally, for both mitotic and apoptotic sequences, no significant differences were observed in the cell-cycle channel expression, which marks the progression through the specific cell-cycle phases. This suggests that cell-cycle progression was not strongly associated with the features emphasized by the model in this analysis.

To assess whether the aforementioned features depend on the experimental condition, we repeated the same analysis separately for the control condition and each of the three cisplatin doses (S4 Fig). Since untreated cells undergo apoptosis more rarely, the control condition contains only 41 apoptotic sequences (see Table 1) and the corresponding estimates are very noisy. We therefore do not interpret the control apoptotic estimates and restrict the following observations to the cisplatin-treated conditions and, for mitosis, to the control condition.

Across the three cisplatin doses, all features show the same trends of change in the high-attention frames. Several features additionally exhibit a consistent gradient with dose. For mitotic sequences, the size-related effects weaken with increasing dose (area, equivalent diameter, and perimeter), whereas the shape-related effects strengthen (circularity and eccentricity). The p53 effect increases slightly and remains the largest effect throughout. The high-attention signature of division shifts gradually from size-based toward shape-based cues as the dose increases, while its overall structure remains unchanged. For apoptotic sequences, the three treated doses show a comparable mild gradient, with p53, perimeter, area, and circadian effects decreasing slightly from low to high dose.

#### 3.2.4 Feature trajectories.

The preceding analyses summarize the attention distribution (Sect 3.2.2) and the high- vs. low-attention feature differences within sequences (Sect 3.2.3) as aggregate quantities. To complement these, we additionally present the full temporal trajectories of attention weights and of each feature, aligned to the cell event (S5 Fig). This trace-level view shows how each cell feature evolves over the cell’s lifetime and how these dynamics relate to the frames the model attends to for prediction.

The fate-resolved trajectories are consistent with the effect-size analysis. For mitotic sequences, attention is sharply concentrated in the final frames, coinciding with a late increase in cell area and equivalent diameter, a rise in circularity, and a pronounced drop in eccentricity, together reflecting possible mitotic rounding. The p53 channel rises steeply toward the event, mirroring its large positive effect size. For apoptotic sequences, the trajectories change more gradually and over a broader temporal window. Area and perimeter increase while circularity decreases, consistent with cell stretching rather than rounding, and p53 intensity is elevated over an extended period preceding the event rather than only in the final frames. The cell-cycle channel remains comparatively flat over time for both fates.

Comparing the two fates at matched positions relative to the cell event also reveals which features are unique for each of them. The p53 and cell-cycle reporters are markedly higher in mitotic than in apoptotic sequences throughout the observed window, whereas the circadian reporter reaches comparable levels in both fates near the event. The shape features separate the fates in the opposite direction, as apoptotic cells retain a more eccentric, less circular morphology, while mitotic cells become rounder towards division.

We further examined whether these patterns depend on treatment or dose, complementing the condition-resolved effect-size analysis above with the corresponding trajectory view. While the analyses above are restricted to cisplatin-treated cells, here we additionally include the untreated control condition for comparison. The attention profiles and morphological features are largely consistent between control and cisplatin-treated cells (see S6 Fig), indicating that the shape-based cues underlying the model’s predictions are not specific to the treatment. The reporter channels, however, differ between conditions. Circadian intensity in high-attention frames is markedly higher under cisplatin than in control for both fates, and the cell-cycle reporter in apoptotic cells remains elevated in the control condition but is low in treated cells. These differences are consistent with cisplatin perturbing the underlying signaling dynamics, while leaving the morphological progression toward each fate largely unchanged. We note, that the control curves are considerably noisier because of the low number of apoptotic events (see Table 1), and we therefore refrain from drawing strong quantitative conclusions from this comparison.

Across the three cisplatin doses (S7 Fig), the attention and feature trajectories are largely indistinguishable, with only a minor reduction in circadian intensity at the lowest dose.

## 4 Discussion

The ability to predict cell fate from microscopy videos transforms time-lapse imaging from a descriptive tool into a quantitative framework for identifying early determinants of drug response, stratifying heterogeneous populations, and guiding mechanistically informed therapeutic strategies. Here we present our computational approach for cell fate prediction from raw video data of cell trajectories. Our Transformer model exploits not only static visual information but also its temporal dynamics in the prediction. Together with the model we design a thorough explainability pipeline pointing to the video frames where the predictive information is located and to the statistically significant properties of cells in these video frames.

Our first finding is that cell fate can be reliably predicted even ~8 h prior to the cell’s death or division. This prediction is performed on raw video data that encodes cell morphology, presence of three dynamically expressed molecular markers and their change in time. The fact that such input allows us for cell fate prediction with high accuracy suggests that this input provides the right information for this task. Importantly, the model does not depend exclusively on the final pre-event frame but integrates signals from preceding frames throughout the sequence.

Secondly, through a systematic explainability analysis of the model, we point to the video frames and cellular features that are exploited by the model in prediction. By combining aggregated attention distributions, temporal truncation analyses, and attention-guided feature comparisons, we observed systematic differences in how the model processed mitotic and apoptotic sequences. In the following, we discuss these observations in relation to cell fate dynamics and model explainability.

### 4.1 Attention focus

Differences in the temporal distribution of attention weights suggest that the model relied on distinct temporal cues when predicting mitotic versus apoptotic outcomes. In mitotic sequences, attention was almost exclusively concentrated towards the end of the sequence. This indicates that the model primarily leveraged late-stage visual cues to identify mitosis, potentially corresponding to abrupt and short-lasting changes in cell morphology or changes in expression of the imaged proteins that occurred just before division. These changes might involve rounding of the cell and fluctuation of nuclear fluorescence signals during rounding and after nuclear envelope breakdown [22–24]. This is also supported by our results shown in Fig 3d, that indicate that the model already achieved a high recall in mitosis classification based on the last 10 frames only (recall: 0.933).

In contrast, apoptotic sequences exhibited a broader distribution of attention over time. Our analysis suggests that apoptotic cell death is preceded by cellular processes that emerge substantially earlier in time. Rather than concentrated near the end of the sequence, attention was distributed across a wider temporal range, with many frames showing high relevance well before cell death. We hypothesize that predictive information for apoptosis emerges gradually, such that early morphological or intensity changes already contribute to the model’s decision. This interpretation is further supported by the truncation experiment in Fig 3d, which showed that restricting the model to only the final frames was insufficient to achieve strong performance for apoptotic sequences.

### 4.2 Attention-guided morphological features

Using our explainability analysis we point to visual features that allow the model to make a correct prediction. Our systematic, statistical analysis has pointed to mostly morphological features as well as p53 expression that are indicative of the cell outcome. Our method applied to more comprehensive cancer cell imaging data can allow to identify early determinants of drug resistance and sensitivity. Based on the identified features resistant cells can be isolated and used to guide prospective intervention — e.g., by applying secondary drugs, or perturbing signaling pathways at time points when cells are most vulnerable. Such cells can be additionally characterized using, e.g., single cell transcriptomics in the particular time points when their fate is determined. The features identified here could be validated causally by ablating them individually from a features-only model and testing whether performance changes match our importance rankings.

**Mitosis** For cells undergoing mitosis, high-attention frames contained cells with increased size, larger area, equivalent diameter, and perimeter measurements. This growth was accompanied by a decrease in eccentricity together with an increased circularity, indicating that cells progressively expanded, becoming larger and rounder. This transition from elongated to more circular shapes is consistent with known mitotic morphology, where cells undergo rounding prior to division, and aligns with previous observations in live-cell imaging studies ([23,24]). To predict mitosis, therefore, the Transformer primarily relies on morphological cues associated with cell rounding that occur shortly before cell division.

Interestingly, intensity changes in p53 were also highlighted by the model as predictive of mitosis. p53 is frequently located in centrosomes during cell division and acts as a sensor for the mitotic surveillance pathway [25]. Furthermore, p53 plays a role in the detection of mitotic extension, preventing daughter cells resulting from abnormally long mitosis from progressing further in the cell cycle [26]. Among the myriad of functions p53 has, it is plausible that high-attention patterns in mitotic sequences relate to the role of p53 in genomic integrity. Increasing evidence also suggests that p53 signaling dynamics, such as signal duration, amplitude, or accumulation rate, encode biologically meaningful information that influences cellular outcomes [27]. Cells that resume proliferation after DNA damage do this via escape from p53-mediated arrest through characteristic transient changes in p53 activity shortly before division [28]. These findings underline the advantage of our approach, by processing the entire video sequence, our model captures not only snapshots of p53 activity but its dynamics across the cell cycle.

The cell-cycle channel, by contrast, contributed little to identifying mitosis, which may reflect the reporter itself. CFP-hGeminin is expressed throughout S/G2/M rather than being mitosis-specific, so it remains at near-constant levels over much of the cycle. As the last analyzed frame precedes division, and the reporter signal drops only during the metaphase-to-anaphase transition once the APC complex becomes active, the characteristic cell-cycle change associated with mitosis falls outside our analysis window.

**Apoptosis** In contrast to mitosis, the attention patterns associated with apoptotic outcomes were more broadly distributed in time. Rather than pinpointing a consistent temporal ordering of predictive events, attention in apoptotic sequences suggests the presence of informative frames across the cell’s lifespan. Frames receiving high attention were characterized by increased cell size relative to low-attention frames within the same sequence. In contrast to mitosis, however, this increase in size was not necessarily confined to the final frames preceding the event and involved a decrease in cell circularity. These morphological changes involve cell stretching rather than rounding and can occur earlier in a cell’s trajectory.

In addition, high-attention frames in apoptotic sequences exhibited elevated p53 intensity, highlighting p53-related signals as an important contributor to the model’s predictions. It is well established that following DNA damage, p53 signaling is activated and plays a central role in coordinating cell fate decisions, including cell-cycle arrest, DNA repair, and the initiation of apoptosis [29–32]. Sustained p53 activation can occur several hours before an apoptotic event [33,34]. We therefore speculate that prolonged p53-related intensity changes in apoptotic sequences may constitute one of the early cues captured by the model and reflected in the broader temporal distribution of attention.

In summary, by using truncation experiments and explainability analysis we quantify how predictive information accumulates over time with longer observation window. The ability to predict cell death early in a cell’s lifetime reflects the extent to which this fate is pre-determined versus progressively acquired. Our approach enables identification of critical temporal windows during which fate becomes predictable.

## 5 Conclusion

The combination of Transformer models with attention-based interpretability provides a powerful framework for the analysis of complex video data. By operating on full temporal sequences and leveraging the attention mechanism, the proposed framework is particularly well suited to capturing dynamic cellular processes and temporally evolving molecular signals present in time-lapse microscopy data. Our results demonstrate that this approach not only enables prediction of biological phenomena but also allows the extraction and interpretation of the signals underlying these predictions. Particularly in biological systems that remain incompletely understood, the methodology presented here offers a valuable strategy for identifying informative patterns and gaining insight into the mechanistic determinants that drive specific phenotypic outcomes.

## Supporting information

S1 Fig Sequence-length distributions across fate and dose. Distributions of sequence length in frames. **a)** By cell fate. **b)** By cisplatin dose. **c)** By treatment. Cisplatin-treated cells are grouped together.
(EPS)

S2 Fig Distribution of trigger-event times. Histogram of the trigger-event positions, measured in frames prior to the cell event, shown separately for mitotic and apoptotic sequences. Only sequences exhibiting a trigger event are included (1,312 of 1,593 mitotic and 212 of 401 apoptotic sequences).
(EPS)

S3 Fig Truncation analysis on a length-matched subset. Truncation analysis repeated on a subset in which apoptotic and mitotic sequences share the same length distribution and all sequences are at least 50 frames long, so that an identical population is evaluated at every truncation level. **a)** Balanced accuracy as a function of the number of truncated final frames. **b)** Class-wise recall for apoptosis and mitosis under the same truncation.
(EPS)

S4 Fig Effect size analysis by treatment condition and dose. The same analysis as in Fig 7, computed separately for the control condition and each of the three cisplatin doses (low, medium, high). For each feature, the median Cliff’s delta and its 95% confidence interval are shown separately for mitosis and apoptosis. Positive values indicate higher feature values in high-attention frames compared to low-attention frames, whereas negative values indicate the opposite. The background shading denotes commonly used effect size categories (negligible, small, medium, and large effects). Note that the control condition contains only 41 apoptotic sequences, resulting in wide confidence intervals for the apoptotic control estimates.
(EPS)

S5 Fig Attention and feature trajectories by cell fate. Mean attention weight (top panel) and median values of each morphological and intensity-based feature as a function of the number of frames prior to the cell event, shown separately for mitotic (blue) and apoptotic (red) sequences on the cisplatin-treated data. Shaded regions denote the interquartile range across sequences.
(EPS)

S6 Fig Attention and feature trajectories: control versus cisplatin. Mean attention weight (top panel) and median feature values as a function of the number of frames prior to the cell event, shown separately for mitotic and apoptotic sequences in control (dash-dot) and cisplatin-treated (solid) conditions. Control curves are noisier due to the smaller number of events, in particular apoptotic events.
(EPS)

S7 Fig Attention and feature trajectories across cisplatin doses. Mean attention weight (top panel) and median feature values as a function of the number of frames prior to the cell event, shown separately for mitotic and apoptotic sequences at high, medium, and low cisplatin dose.
(EPS)

S8 Fig Example apoptotic sequence. Illustrative apoptotic trajectory with corresponding temporal attention weights. Frames assigned high attention by the Transformer model are highlighted in green, whereas representative low-attention frames are marked in red. Attention weights were normalized across the sequence.
(EPS)
