# Supplementary material for: Understanding cell fate decisions with temporal attention
Source: PLoS Comput Biol. 2026 Oct 12;22(10):e1014813. doi: 10.1371/journal.pcbi.1014813 (PMC13657290; doi:10.1371/journal.pcbi.1014813)
Supplement: Supplementary file 1 [file pcbi.1014813.s010.pdf]

Revision for

Understanding cell fate decisions with temporal attention

Reviews

Additional Editor Comments

As discussed by all reviewers, this work is well-motivated and interesting, especially regarding the extensive analysis on temporal information. However, reviewers raise concerns about the details on methodology and experimental results for supporting the conclusion. For the lack of open-source code and data, the reproducibility of this work is also limited for future work. We require authors to conduct substantial revision according to the comments from all reviewers.

- We thank the editor and reviewers for their thoughtful assessment of our manuscript. We greatly appreciate the constructive comments and suggestions, which have enabled us to strengthen the manuscript and validate our approach more thoroughly.
- Both the source code and the dataset are publicly available. The source code is provided at `https://github.com/bozeklab/Cell-Fate-Prediction`, and the raw dataset is published at `https://doi.org/10.4126/FRL01-006526608`. These links are included with the submission, and we restate them here to ensure they are accessible to the reviewers.

In the following, we give a point by point response to the reviewers' comments.

Review 1

Overall assessment:

In this manuscript, authors applied a deep learning based computational approach to model the longitudinal series of single-cell images in different settings: control versus cisplatin treatment. With the goal of predicting mitosis against apoptosis, authors discussed the effectiveness of their modeling approach and further analyzed important contributors identified by the approach. Overall the concept and study design are very clear, but the datasets, methods, and interpretability analysis would need some further clarification and justifications. Please see below a full list of major and minor concerns:

Major:

1. The dataset used for the study could be further clarified:
   (a) In total 51k sequences are curated, out of which only 21.5k are annotated. Are the remaining trajectories used in any form during the training?
      - To avoid confusion, we have revised the terminology in the manuscript. Of the 51k curated sequences, all were annotated with cell centroid position and id; however, only 21.5k were annotated with cell fate of either mitosis or apoptosis, which are the sequences used for the supervised classification task presented here. The remaining trajectories did not carry one of these two terminal-fate labels (e.g., cells left the field of view before a fate could be determined or the video ended before the respective cell fate) and were therefore not used in the training of the binary classifier. We have clarified this distinction in the revised manuscript (line 115-118).
      - Moreover, in accordance with other reviewer's comments, we included a multi-class classification that includes these sequences (line 287-297).

   (b) How are the trajectories labeled? The quantity here seems a bit unrealistic for human labeling, may authors disclose how the labels are acquired? Also is the task a binary classification?
      - The labels were not assigned in a binary manner at annotation time. Instead, each cell was tracked manually by marking its centroid across consecutive frames, with mitotic and apoptotic events recorded at the frame of division and the first frame showing clear signs of cell death,

respectively. Therefore each cell is manually tracked and its fate is labeled which represents massive manual effort involved and reflects the scale of the dataset. From these annotations we derived the terminal-fate labels used here. The task addressed in this paper is a pure binary classification problem, distinguishing mitosis from apoptosis. We have added the annotation procedure to the revised manuscript (line 109-118).

(c) There is no example image/video in the figures, I would recommend include some example cases.

- We agree that example cases improve the clarity and accessibility of the work. In addition to the exemplary mitotic sequence already shown in the initial submission (Fig. 8), we have added an exemplary apoptotic sequence with its corresponding attention weights (Fig. S8), so that representative frames are now shown for both fates.

(d) From what I understand, the dataset is cell culture, and the cell might migrate in the well. How did authors track the cells across timepoints? Would there be basic metrics to show that this step is done properly? And how would tracking errors propagate to downstream modelings of cell fate?

- In the described experimental setup of this manuscript, cells are adherent and imaged under standard culture conditions without any chemo-attractant, wound, or other directional migration cue. Net cell migration across the field is therefore minimal over the imaging period. Cells that do leave the field of view are excluded from the analysis, as noted in Sec. 1.1.
- In this manuscript, we do not employ an automated cell-tracking algorithm to extract the trajectories. The trajectories are derived directly from manual human annotations, in which each cell's centroid is marked in every frame. The model input is a center-crop around these annotated centroids. Thus, there is no algorithmic tracking step in our pipeline, and, therefore, none of the failure modes characteristic of automated tracking. The only potential source of trajectory error is therefore annotation error. To control annotation reliability we defined a standardized annotation protocol with shared event definitions. We also reviewed and corrected randomly selected portion of the annotations and lineage assignments. That is why standard multi-object tracking metrics are not applicable, as no tracking algorithm was used here. Via our annotation protocol and review described above we ensured that the trajectories are possibly error-free.

(e) Does the dataset contain bright field images? From examples in Fig. 6, it seems that morphology beyond just cell shape is not well captured by these fluorescence channels.

- The dataset does not contain bright-field images. The recordings consist solely of the three fluorescence channels (p53, circadian, and cell-cycle reporters). Consequently, morphological information is limited to what these reporters reveal, and finer subcellular morphology not captured by the fluorescent markers is indeed not available.

2. The modeling choice: CNN for embeddings + ViT on sequence of embeddings would need some justification:

(a) Why is a ResNet model used and trained from scratch in this project. Given the model size, there could be a high chance of overfitting. Using some kind of pre-trained models for cellular imaging seems more robust.

- We compared a ResNet-50 backbone trained from scratch against an ImageNet-pretrained ResNet-50 of identical architecture, keeping all other components and training settings fixed. The two configurations yielded comparable performance, with no meaningful advantage for the pretrained initialization. We attribute the limited benefit of pretraining to the substantial domain gap between natural RGB images and our three-channel fluorescence microscopy input, whose statistics and semantics differ significantly from ImageNet.
- Regarding overfitting, we did not observe evidence of it in either configuration. We therefore opted for the from-scratch backbone as the simpler choice that performs on par with the pretrained alternative.

(b) It would be preferable to show at least some comparison against naive baselines, such as heuristic morphology (cell size, aspect ratio, etc.) based on segmentation, averaged across the sequence or to replace the CNN embeddings.

- Following the reviewer's suggestion, we implemented a morphology-based baseline. We segmented cells with Cellpose and extracted the same handcrafted per-frame features as used in our intepretability analysis. These per-frame feature vectors were passed through a small MLP

to produce cell embeddings in each frame, which then replaced the ResNet-50 embeddings as input to the same Transformer encoder. The only architectural change was the Transformer's input embedding dimension, adjusted to match the feature-embedding output rather than the ResNet-50 output. All other components and training settings were kept identical, so that only the input representation differs.

- This baseline was able to discriminate the two fates but at reduced performance, achieving a BAcc of 0.90 and an F1-score of 0.877, compared to 0.94 and 0.93 for the full model operating on raw image sequences. We have added this baseline and the comparison to the revised manuscript (line 170-181 and line 280-286).

(c) Are the paddings for shorter sequences before or after the sequence? Since positional encoding is used here to indicate timestamp. It makes sense to label the event time (for mitosis/apoptosis) uniformly across sequence, so the "positions" here are more like T-1, T-2, etc. Can authors disclose some more details on the calculation of positional encodings and paddings?

- During training, sequences within a batch are padded at the end to a common length. The padded positions are masked within the Transformer encoder so that they do not contribute to the attention computation (Sec. 1.4). Sinusoidal positional encodings are computed on absolute frame indices, starting at the first appearance of the cell. At evaluation time, sequences are processed individually, so no padding is applied at all and every sequence is processed at its true length. The reported results are therefore unaffected by the padding scheme. We have made the indexing of the positional encodings and the absence of padding at evaluation explicit in the revised manuscript (line 158-160, line 235-236).
- Regarding the concern that the position of the cell event varies across sequences, we address this during training through a sequence-level augmentation in which a contiguous segment at the end of each trajectory is masked, with the masked portion ranging between 10 and 50% of the sequence length (Sec. 1.4). This prevents the model from training on sequences where the fate event is at a fixed position, and it is what makes the truncation experiments in Sec. 2.1.1 meaningful, as the model has been trained to handle trajectories that end at varying distances from the fate event.
- We agree that event-anchored positional encodings are reasonable alternative design, which we did not adopt here. Our results indicate that the model nonetheless learns fate-relevant temporal structure from absolute encodings, as demonstrated by the attention and truncation analyses.

3. Are the surrounding cells in the image patches masked? If not, would they affect/confound the model predictions? For example, cells in densely populated area are more likely to undergo apoptosis/mitosis.

   - The surrounding cells in the image patches are not masked. We provide the center-cropped patches as they are, and the model receives no annotation of the fate or identity of any neighboring cells that may be partially visible. We agree this is an important point regarding potential confounds.
   - However, two aspects of our setup limit the influence of neighborhood context. First, the patches are center-cropped tightly around the target cell, so that only a small number of immediately adjacent cells fall within the field of view. The crop does not capture the broader local density or the structure of the wider neighborhood, and the model therefore does not have access to the population-scale density information that would be needed to exploit cues such as cells residing in densely populated areas. Second, because no fate or label is provided for any neighboring cell, the model cannot directly tie its prediction to the outcome of surrounding cells.
   - In one of our analyses, we explicitly accounted for the immediately surrounding cells by quantifying the neighbor count around each target cell as a proxy for local density, and tested whether this measure was predictive of the respective cell events. We did not find any significant effect, further suggesting that neighborhood context is not a primary driver of the model's predictions.

4. Are distributions of sequence lengths different between mitosis and apoptosis? And between control / cisplatin-treated samples, would there be differences in class balance, distributions of sequence lengths, average morphology, etc.?

   - We have added a characterization of the sequence-length distributions to the revised manuscript (Data section, line 131-138, Fig. S1).

- Sequence lengths differ markedly between the two fates: apoptotic sequences are considerably longer than mitotic ones, reflecting the different time scales of the two processes (Fig. S1a). Between control and cisplatin-treated cells, the median sequence length is similar, but treated cells show a broader distribution with a longer tail toward high sequence lengths (Fig. S1b, Fig. S1c). Across the three cisplatin doses, the length distributions are broadly comparable, with only a slight decrease at lower doses.
- Regarding class balance, the composition of the dataset by fate and dose is now reported in Table 1 (Sec. 1.1, line 139-147). The proportion of apoptotic events increases with dose, while the proportion of mitotic events decreases correspondingly.
- Regarding morphology, we now report the temporal trajectories of all morphological and intensity-based features separately for control and cisplatin-treated cells (Fig. S6, Sec. 2.2.4). The morphological features are largely consistent between the two conditions, whereas the reporter channels differ. We additionally repeated the high- versus low-attention feature analysis separately for the control condition and each cisplatin dose (Fig. S4, Sec. 2.2.3), which shows that the feature contrasts share the same tendencies across doses.
- We note two aspects of the data composition that are relevant for the downstream analyses. First, the control condition contains very few apoptotic events (41 sequences). This is why our interpretability analyses focus on the cisplatin-treated sequences, where apoptotic dynamics can be characterized with adequate sample size. Second, a large subset of sequences originates from videos for which the administered dose is unknown. As no dose information is available for these cells, they are excluded from dose-stratified and treatment-specific analyses, which accounts for the reduced sequence counts in those parts of the manuscript.

5. The interpretability part seems to be a highlight of the manuscript, but some aspects could be further polished:

   (a) Fig. 5 shows some features captured in the "important" frames, can we further examine their directionalities towards the prediction of mitosis/apoptosis? For example, does high p53 mean higher chance of apoptosis?

   - We thank the reviewer for this suggestion.
   - We first note that the feature importance in Fig. 5 (initial submission) is computed within each fate class. It compares high-attention against low-attention frames of the same sequences, and therefore characterizes which features the model attends to, separately for mitosis and apoptosis. It does not indicate whether a feature points toward one fate rather than the other, which is the directionality the reviewer raises.
   - To address this, we compare the features between fates at matched positions before the event, using the frame-aligned trajectories (Fig. S5, Fig. S6, and Fig. S7). Regarding the specific example raised by the reviewer, elevated p53 does not indicate a higher chance of apoptosis. At matched positions, p53 levels are in fact considerably higher in mitotic than in apoptotic sequences, as is the cell-cycle reporter, whereas the circadian reporter reaches comparable levels in both fates near the event. The shape features separate the fates in the opposite direction, with apoptotic cells retaining a more eccentric, less circular morphology while mitotic cells round up before division.

   (b) Since each sequence contains longitudinal data points, it is expected that frame t might contain lots of similar information as frame (t-1) and (t+1). The strategy of using attention weights as feature importance might not be able to natively capture this longitudinal continuity, may authors comment on their choice here?

   - The reviewer is correct that adjacent frames can be redundant, and that attention magnitude on a single frame should not be read naively as a frame-level importance score.
   - We address this in several ways, First and most importantly, redundancy is limited by our 30-minute sampling interval. Consecutive frames can differ substantially, and key transitions are abrupt rather than gradual, e.g., mitotic rounding typically appears within the final one or two frames, where the attention profile peaks sharply.
   - Furthermore, we do not rely on attention weights alone. Our masking experiments (Fig. 3, initial submission) measured importance causally by removing frames and observing the effect on prediction. Removing high-attention frames degrades performance more than removing random

or low-attention frames (cf. new Fig. 5). This would not occur if their information were fully redundant with neighboring frames.

- Lastly, our analysis operates on the Top-10% set of frames per sequence ($\mathcal{H}_s$), aggregated, rather than on isolated single frames, so predictive information spread across a block of similar frames is captured collectively rather than diluted.

(c) It is also widely recognized that attention weights could be arbitrary. In addition to this attention-based importance scores, would there be alternative measures to get frame importance? A naive idea would be to mask certain frames (just like what authors did in Fig. 3) and see how such masking lead to changes in predictions.

- We thank the reviewer for this suggestion. This aligns closely with a point raised by another reviewer, and we have addressed both with a new masking experiment (Sec. 2.2.1, new Fig. **??**, line 391-396). For each sequence in the test set, we mask an increasing fraction of frames and measure the resulting change in prediction, comparing three selection strategies: masking the highest-attention frames, the lowest-attention frames, and the same number of randomly chosen frames. This provides a causal, attention-independent measure of frame importance. Masking the high-attention frames degraded performance markedly more than masking random or low-attention frames across all fractions, corroborating the attention-based importance score.

6. Since the key topic of this manuscript is about cell fate, I think a key type of discovery is some kind of trigger events detected by the sequence transformer that heavily affect the predictions of cell fate. Authors have only discussed these qualitatively ("rounding of the cell and fluctuation of nuclear fluorescence signals"), but I would recommend extending the masking experiments in Fig. 3 to further reveal this type of information. For example, given a sequence, can we show the curve of predicted mitosis/apoptosis along the sequence, where each point's prediction is only based on the history observed until this point. A sharp change in this curve could be indicative of a trigger event.

   - We agree that this is a valuable complementary view and have implemented the suggested analysis. For each cell, we calculated the predicted event probability from the observed history from the first frame up to frame t, increasing t towards the event. This yields a per-sequence prediction curve in which each point depends only on the frames observed so far. We define a trigger event as the last crossing of the 0.5 decision boundary along this curve. We search for this crossing only from the $10th$ frame onward, since predictions based on very few initial frames are unreliable and would otherwise produce spurious crossings. This restriction applies only to where trigger events are detected, the earlier frames were still used by the model for prediction at every subsequent step.
   - We would like to point out that this analysis is related to, but distinct from, the truncation experiments already reported in Fig. 3. The latter vary the amount of observed context and report aggregate performance, therefore answer how much temporal context is required on average. The suggested analysis instead resolves individual trajectories and localizes the frame at which the model commits to a fate.
   - Restricting the analysis to the 1,994 correctly classified sequences, we find a marked asymmetry between the two fates. 80% of mitotic sequences exhibited a trigger event, with crossings occurring most frequently 20–35 frames prior to division, whereas only 48% of apoptotic sequences did, with crossings distributed across the entire observed window without a preferred temporal position. The remaining sequences were assigned to the correct class from the earliest evaluated prefix onward and never crossed the decision boundary. This is consistent with our observations that apoptotic fate is determined comparatively early, whereas the fate of mitotic sequences is decided shortly before the event. The analysis has been added to the revised manuscript (lines 342-349), with representative trajectories in Fig. 4 and the distribution of trigger frames in Fig. S2.

7. Would there be any differences between control / cisplatin-treated cases in terms of their interpretability results? Given the effect of cisplatin as a chemotherapy reagent to trigger apoptosis, it can be imagined that the important frames might show different distributions as compared to control cases. Discussion on such differences is missing.

   - We thank the reviewer for this suggestion. In response to this and a related comment from another reviewer, we have added Fig. S5, Fig. S6, and Fig. S7 showing the attention profiles and morphological feature trajectories separately for control and cisplatin-treated cells.

- For mitotic sequences, the attention profiles are similar between the two conditions, with attention concentrated sharply in the frames immediately preceding the division in both cases. For apoptotic sequences, however, the profiles differ. In cisplatin-treated cells attention rises progressively starting approximately 40 frames before the event, whereas in control cells attention is more uniformly elevated across the trajectory without a comparable rise. This is consistent with the mechanism the reviewer suggests. Under cisplatin, apoptosis is induced by the drug and the model's attention appears to track the onset of the resulting death process, while spontaneous apoptosis in untreated cells lacks a comparable temporally localized trigger, so no particular window is privileged.
- We additionally repeated the high- versus low-attention feature analysis separately for the control condition and each cisplatin dose (Fig. S4, Sec. 2.2.3). For mitotic sequences, the features are consistent across all four groups, indicating that the features the model attends to to predict division are not specific to the treatment.
- Since untreated cells undergo apoptosis more rarely, the control condition contains only 41 apoptotic sequences, compared to approximately 2,200 in the cisplatin-treated conditions. Therefore the features extracted from the apoptotic sequences in the control condition are noisy and inconclusive. This limited sample size, together with the fact that spontaneous and drug-induced apoptosis are mechanistically distinct processes, is why our analyses focus on the cisplatin-treated conditions, where the question of what determines divergent fate under chemotherapeutic treatment can be addressed with adequate statistical power. We have added it to the revised manuscript (Sec. 2.2.4, line 498-539).

8. In practice, it would be nearly impossible to get continuous measurements out of a real cancer patient's tumor. In cases where only snapshots can be acquired (high-dimensional spatial omics, for example), may authors discuss how this proposed modeling approach/concept can be applied or adapted?
    - We agree with the reviewer that continuous measurements from a real patient's tumor are generally infeasible, and that in practice only snapshots (e.g., high-dimensional spatial omics) are available.
    - We propose that an approach such as ours can however be instrumental in identifying cell populations and their dynamic features that allow them to resist chemotherapeutic treatment. Identification and further in-depth study of these cell populations can help to discover resistance mechanism of cancer that might be not uniquely genetic but also be related to their dynamical states and timing of the treatment.

Minor:

1. Fig. 4 is a bit hard to read, maybe use a scatter/line plot instead.
    - In accordance with this suggestion and another reviewer's comment, we changed the plot to a line plot with bands showing the deviation (new Fig. 6).
2. Line 170-174: "Across all sequences, this results in n p-values and n corresponding Cliff's delta values ..." What is 'n' here? Do you mean "across ONE sequence" or actually across all sequences of the dataset? I find this paragraph a bit hard to understand.
    - 'n' means the number of sequences that were used for the respective analysis. Since across our experiments, this 'n' changes based on the dataset (Cisplatin-treated sequences only, control sequences only, etc.). We updated that paragraph and explained the 'n' in more detail (line 219-225).

Review 2

Overall assessment:
Bürger et al., has developed a Transformer-based model that predicts single-cell fate directly from raw time-lapse microscopy sequences of cisplatin-treated cancer cells, reporting a balanced accuracy of 0.94. They introduce an explainability pipeline that finds high attention frames and by using a Cliff's-delta comparison they investigate which morphological or intensity-based features leads to the high attention frames. Here they find that the p53 channel showed a pronounced positive effect.

In my opinion, the problem is well-motivated and investigates a very deep question. The main results and conclusions are very interesting though vital aspects remain unclear. The dataset applied for this analysis is small compared to normal ML approaches (which is not a bad thing) but good enough to draw conclusions. The writing of the paper is very direct (which again is not a bad thing) for the most part clear. Therefore I do

see that this work can be published in Plos comp. Biol, however I have however a series of questions and points where I believe the authors do not support their main conclusions and to publish this paper would therefore require a major revision.

General problem: The authors report very profound results but I believe the evidence of these are often thin. First of all, the two central claims of the paper: 10 h predictability and importance of p53 channel is not supported directly by a figure. I really need the authors to make this more clear.

- We thank the reviewer for the positive and constructive feedback. We address these points in detail in the answers below and support our results with new figures.

Major:

1. Figures: I would very much like more figures showing the dynamical traces of many of these observables - in particular p53 and circadian information. Also I need figures (histograms at least) that outline what makes the high attention weights dominating (see my point E on this also). Also, there should be a qualitative apoptotic example analogous to mitosis in Fig. 6. Furthermore, Fig. 4 is really hard to read quantitatively; the numbers quoted in text (0.62, 0.22, 0.88, 0.42) deserve a line plot with confidence bands, not a grayscale strip.
    - We thank the reviewer for these suggestions, which we have addressed as follows:
    - We have added new figures (Fig. S5, Fig. S6, and Fig. S7) showing the temporal trajectories of the morphological and intensity-based features, including p53 and circadian channels, alongside the attention profile, so that the dynamics of these observables can be inspected directly and related to the frames the model attends to. These trajectories show that the p53 signal follows distinct temporal profiles in the two fates, rising steeply in the final frames preceding division but elevated over a broader window in apoptotic sequences. Shape features reflect the pronounced rounding of mitotic cells against the more eccentric morphology retained by apoptotic cells.
    - To characterize why the high-attention frames drive the prediction, we have added a masking analysis (Fig. 5). This shows that removing high-attention frames degrades the performance substantially more than removing random or low-attention frames, establishing causally that these frames carry the information the model relies on.
    - We have added a qualitative apoptotic trajectory analogous to the mitotic example, with high- and low-attention frames highlighted in the same manner (Fig. S8).
    - We have replaced the initial Fig. 4 with line plots showing the median attention profile together with confidence bands, so that the quoted values can be read quantitatively (new Fig. 6).

2. Regarding the 10 h claim, then I cannot trace it to a specific quantitative result. In Fig 3a, the authors show BAcc stays above approximately 0.9 up to 20 frames removed (which means 10 h) - but this definition should be more clearly stated. Also, this is overall BAcc on a set dominated by mitosis, and Fig. 3c shows mitotic recall already drops at 5 frames. I therefore understand this as the 10 h statement holds mainly for apoptosis, not as a general claim? Please describe this result in greater detail and state the claim per-class, define it against a stated baseline, and mark the relevant threshold explicitly on Fig. 3.
    - We have revised both the analysis and its description and provide a more detailed and precise description of this result in the revised manuscript.
    - We have re-run the truncation analysis on the full test set rather than the cisplatin-treated subset. On the full test set, overall BAcc remains close to the untruncated baseline (0.94) up to a truncation of 15 frames (0.903) and declines more steeply thereafter. We therefore changed our claim from 10 hours (20 frames) to approximately 8 hours (15 frames).
    - We now report the result per class, as the reviewer requests. At a truncation of 15 frames, both classes' recalls remain above 0.9 (apoptosis: 0.9; mitosis: 0.900), so the claim holds for both cell fates. Beyond this threshold the two classes diverge: apoptotic recall remains stable, whereas mitotic recall declines steeply, reaching 0.254 at a truncation of 50 frames. We note that on the previously used cisplatin-treated subset, mitotic recall declined earlier, which is why the reviewer's reading of the original figure was justified; on the full test set at the revised threshold, the claim is supported for both fates individually.
    - Finally, we have marked the relevant threshold explicitly on Fig. 3 with a vertical line at 15 frames, so that the claim can be traced directly to the figure.

3. I find the p53 claim very interesting but was disappointed with the (lack of) evidence revealed in the paper. In the discussion, the authors discuss how: "the distinct temporal attention profiles for mitotic versus apoptotic sequences mirror the distinct p53 dynamical states that mechanistic studies have identified as fate-determining", citing work of the Lahav lab. However, the authors show no traces of neither p53 or circadian information and in the main text they write that high-attention frames have elevated p53 intensity on average. There is no analysis of p53 dynamics despite the authors explicitly invoking these as the mechanism. I therefore see two options: either add an analysis that directly tests a dynamical hypothesis based on p53 trajectories and ask whether a dynamical feature (pulse count, slope before event) separates fates and aligns with high-attention frames or substantially downtune the claims of mechanisms and explainability claims throughout the text. As written, the interpretation is largely overstated compared to the evidence revealed.

   - We have added the temporal trajectories of the morphological and intensity-based features, including the p53 and circadian channels, shown separately for the two fates (Fig. S5, Sec. 2.2.4). These traces show that the p53 signal follows distinct temporal profiles in the two fates. It rises steeply in the final frames preceding division, whereas in apoptotic sequences it is elevated over a considerably broader window preceding the event. Between fates, p53 levels are markedly higher in mitotic than in apoptotic sequences at matched positions.
   - We agree, however, that these observations characterize the temporal profile of the signal but do not constitute a test of a specific dynamical hypothesis, such as whether pulse count or pre-event slope separates the fates. We have therefore removed the claim that our attention profiles mirror the distinct p53 dynamical states identified in mechanistic studies, as we do not test this correspondence directly. The discussion now refers to established p53 biology only as context for a explicitly speculative interpretation, and the remaining statements are restricted to what our analyses support, namely that elevated p53 characterizes the frames the model attends to and that its temporal profile differs between fates.

4. A main claim of the paper is that temporal attention matters and to validate this the authors should perform a non-temporal baseline. In particular Fig. 3d shows mitosis can be estimated approximately 0.94 recall from the last 5 frames alone - this could suggest that a static last-frame classifier might already do well for mitosis? This needs to be thoroughly discussed.

   - We thank the reviewer for this suggestion, which turned out to be particularly valuable. Following it, we trained a non-temporal baseline, a ResNet-50 classifier operating on the last frame of each sequence alone. Notably, this baseline showed a larger performance drop for mitosis than we expected, given that mitotic rounding usually happened in the very last frame. This counterintuitive observation prompted us to examine the temporal truncation results in finer detail.
   - This finer analysis (now included in Fig. 3b, Fig. 3d) shows, when the model is restricted to only the last $k = 1$ frames, mitotic recall drops sharply (to 0.641), and it recovers to above 0.9 only once approximately the last $k = 4$ frames are included. In other words, a single frame is not sufficient for reliable mitosis prediction. The model requires a few preceding frames to place the final frame in context. We interpret this as the model recognizing mitotic rounding as a change relative to the cell's earlier shape, rather than from the last frame in isolation.
   - This clarifies why a static last-frame classifier shows lower performance compared to the transformer. To predict mitosis the model requires temporal information that show the rounding process in the final frames and the immediately preceding ones, rather than static frames of round cells. We have added the fine-grained per-frame analysis to the revised manuscript (line 359-375) and believe it addresses the reviewer's underlying concern whether temporal modeling is necessary more directly than a static baseline would.

5. The second half of the paper (Sec. 2.2 and the p53/circadian/morphology interpretation in the Discussion) rests on a critical assumption: that the frames receiving high attention weights are the frames the model actually relies on for its prediction. I am no expert in the current developments in ML, but it is my impression that based on papers by (Serrano, S., & Smith, N. A. (2019). Is attention interpretable? and Jain, S., & Wallace, B. C. (2019). Attention is not explanation - and counter argument Wiegreffe, S., & Pinter, Y. (2019). Attention is not not explanation) it seems to me that this could be validated even further. My simple suggestion for the authors is to show a figure where you take the correctly classified sequences and measure how much prediction degrades when you remove frames, comparing

three conditions: 1) masking the high-attention frames, 2) masking the same number of randomly chosen frames and 3) masking the same number of low-attention frames. If attention is faithful, masking H_s should hurt prediction substantially more than masking random or low-attention frames. If the three are comparable, the attention weights are not tracking importance. In my opinion this would strengthen the central claim of this paper.

- We thank the reviewer for this thoughtful comment. We have implemented the proposed experiment. For all classified sequences in the test set, we progressively mask an increasing fraction of frames and compare three conditions: masking the high-attention frames, low-attention frames, and the same number of randomly chosen frames (Sec. 2.2.1, new Fig. 5, line 391-411). The results support the faithfulness of the attention weights: masking high-attention frames degrades performance substantially more than the other two conditions across all masking fractions. Moreover, low-attention masking consistently degrades performance the least, even less than random masking, further indicating that low-attention frames are the least informative frames for the model.

Minor:

1. The reported embedding dimension is "2024-dimensional" (line 129). Do you mean 2048 for ResNet-50?
   - We in fact meant 2048 dimensions for ResNet-50. We corrected the typo (line 155).
2. BAcc on the cisplatin-only test set is 0.958 (line 243) vs. 0.94 on the full test set (line 215). Please clarify which is the main number and why they differ.
   - The 0.958 value originated from evaluating the truncation experiment on the cisplatin-only subset, while 0.94 is the performance on the full test set. To resolve this and to keep the classification based results consistent, we have re-run the truncation analysis (and the additional classification experiments added during revision) on the full test set. All classification performance is therefore now reported on the full test set, with a balanced accuracy of 0.94 as the single main number. The 0.958 value no longer appears in the manuscript.
3. Cisplatin was given at three dose levels (line 101). Are temporal/attention patterns dose-dependent? Pooling across doses may mask heterogeneity relevant to the overall fate claim. At minimum, report the dose composition of the apoptotic vs. mitotic sets.
   - We now report the dose composition of the apoptotic and mitotic sets in Table 1. As expected from the pharmacology of cisplatin, the proportion of apoptotic events increases with dose, from 0.9% of events in the control condition to 27.4% at the highest dose, while the proportion of mitotic events decreases correspondingly.
   - To test whether the temporal and attention patterns are themselves dose-dependent, we added dose-resolved trajectory analyses (Fig. S7, Sec. 2.2.4). The attention and feature trajectories are largely indistinguishable across the three doses.
   - We additionally repeated the high- versus low-attention feature analysis separately for each dose (Fig. S4, Sec. 2.2.3). All features share the same tendencies across doses. Several features additionally exhibit a mild, consistent gradient. For mitotic sequences the size-related effects weaken with increasing dose while the shape-related effects strengthen, with p53 remaining the largest effect throughout. Pooling across doses therefore does not appear to mask relevant heterogeneity for the patterns we report.

Review 3

Overall assessment:

In this paper, the authors analyze an existing live-cell imaging dataset obtained upon treatment with cisplatin, a common chemotherapeutic agent, in which treated cells, followed for hours, were annotated as mitotic or apoptotic. In this dataset, nuclei, p53 levels, cell-cycle progression, and circadian progression can be monitored through the use of appropriate reporters. By applying a transformer model, the authors are able to accurately classify mitotic and apoptotic cells. Furthermore, attention and masking allow them to identify the variables that contribute most to the classification, with the potential to provide insights into the biological determinants of cell fate decisions.

Using machine-learning methods able to extract features and potentially biological insights into what determines cellular outcomes from live-cell imaging data can certainly be very useful. At the same time, a careful evaluation of the precise advantages and disadvantages of each methodology, and of the extent to which the systems used can really provide biological insight, is necessary. At this stage, this paper does not address these two important issues in a convincing way. To do so, several points must be addressed so that the contribution and value of the present work become evident.

1. The authors declare that their dataset comes from a system developed by some of them called "TransientTrack" (https://arxiv.org/html/2512.01885v1). Inspecting this work, it is evident that it is a machine-learning-based system that is also able to track and classify cells. At this point, it is not clear to me whether the annotations used in the present study were generated with this method. Furthermore, it would be important to understand how the classification performed here compares to that performed by TransientTrack (which is only possible if both are assessed against an independent manually annotated dataset). More details on how the annotations were generated would be necessary to properly assess the biological potential of the method and to ensure that there are no experimental biases.

   - The annotations used in this manuscript are manual ground-truth annotations, not classifications produced by TransientTrack. TransientTrack is a tracking-and-classification method that was trained on and evaluated against this same manually annotated ground truth. However, it is not the source of the labels. Both, this manuscript and TransientTrack rely on the same human-generated annotations as their ground truth, but for different tasks. TransientTrack uses them to train and benchmark a tracking pipeline, whereas here we use them directly as supervisory labels for cell-fate prediction. No TransientTrack predictions were used for our training or evaluation.
   - To make this more explicit, we have expanded the Dataset section (line 109-118) to describe the manual annotation procedure in detail, adapting the relevant description from the dataset publication.
   - Furthermore, a direct comparison of the fate classification (especially for apoptosis) is not straightforward. TransientTrack's fate detection is evaluated only after predicted tracks have been matched to the ground-truth tracks. This matching step is itself a source of error, and the resulting fate-detection performance therefore conflates tracking and matching accuracy with the fate classification itself. In contrast, our model classifies the fate without any preceding tracking or matching step. A head-to-head comparison of the two fate classifications would thus not be feasible, as it would attribute tracking- and matching-induced errors to the fate-classification task.

2. A method able to automatically classify cells undergoing apoptosis, mitosis, or neither of these two outcomes would be of great interest, given the growing availability of live-cell imaging experiments and datasets. It would therefore have been worthwhile to test whether the proposed method was able to classify cells into these three classes: apoptotic, mitotic, or neither. Such a classification should also be compared against TransientTrack.

   - We thank the reviewer for this suggestion. We have trained and evaluated the model in a tree-class setting, distinguishing apoptotic, mitotic, and "neither" cells, and report the results in the revised manuscript (Sec. 2.1, line 287-297, Fig. 2c). The model achieves a BAcc of 0.8434 and a macro F1-score of 0.7946 in this setting. As discussed there, the "neither" class is fundamentally different from the two terminal-fate classes, as these cells have not yet undergone a fate transition, but their eventual fate is unobserved rather than absent. A substantial fraction would, given a longer observation window, go on to divide or die, so this class inevitably overlaps with the two original classes.
   - Regarding a comparison against TransientTrack, we believe such a comparison would not be meaningful. As detailed in our response to the reviewer's first comment, TransientTrack is not the source of our labels: both methods rely on the same manual ground-truth annotations, but in different roles. TransientTrack fate detection is scored only after predicted and ground-truth tracks are matched, a step that introduces its own errors, whereas our model classifies pre-cropped single-cell trajectories directly.

3. A major hurdle in convincing the reader of the power of the method is that it is validated on only one experimental condition: cells treated with cisplatin. Applying it at least to untreated versus treated cells would have provided more convincing evidence of both the validity of the method and the biological insights it can provide. For example, some evidence points to a role of p53 accumulation in cell death

(PMID: 27062928) and to p53 bursts during the cell cycle (PMID: 20598361). I would expect these behaviors to change and produce different patterns in treated cells compared to untreated cells. This aspect is unfortunately unexplored.

- We would like to first clarify that the model itself is not restricted to a single condition. Training and evaluation are performed on the full dataset, which includes untreated and control cells as well as all cisplatin dose levels. The restriction to cisplatin-treated sequences applies only to the interpretability analyses due to the small number of apoptosis events among the non-treated cells.
- We have added a direct comparison of the attention and feature trajectories between control and cisplatin-treated cells to the revised manuscript (Fig. S6, Sec. 2.2.4). The morphological features and attention profiles are largely consistent across conditions, whereas the reporter channels differ. p53 intensity is higher under cisplatin for both fates, and the cell-cycle reporter in apoptotic cells remains elevated in control but is low under treatment. The direction of the p53 difference is consistent with damage-induced p53 accumulation, although the difference is only marginal in apoptotic sequences.
- We have additionally repeated the high- versus low-attention feature analysis separately for the control condition and each cisplatin dose (Fig. S4, Sec. 2.2.3). For mitotic sequences the features are consistent across all four groups. The p53 channel shows a large positive effect and the morphological features retain the same directions and comparable magnitudes irrespective of treatment, indicating that the features the model attends to for division are not specific to the chemotherapeutic condition. For apoptotic sequences, the results among the three cisplatin doses agree closely, whereas the control estimates carry wide confidence intervals due to the small number of apoptotic events in the untreated condition (41 sequences, Table 1).
- These analyses characterize the temporal profile of the p53 signal and how it differs between conditions and fates. We acknowledge, however, that they do not test the specific dynamical quantities examined in the cited work. Our trajectories are aggregated across cells, which by construction obscures the discrete p53 pulsing described by Loewer et al. (2010), and we do not extract per-cell dynamical features such as the accumulation rate or onset time used by Paek et al. (2016) to relate p53 dynamics to death. A dedicated single-cell analysis of p53 pulsing and accumulation kinetics across treated and untreated conditions would be required to test these hypotheses, which we consider a valuable direction for future work.
- Consistent with our response to the preceding comment, we have downtuned the mechanistic claims accordingly.

4. The classification results shown in Figure 4 are striking and deserve further comment. From the results, it appears that cells treated with cisplatin are predicted to be apoptotic very accurately even when the last 50 frames are removed. This roughly corresponds to 25 hours, which is typically the time required for cells to die upon cisplatin treatment according to PMID: 27062928. A point that the authors need to clarify is the exact moment at which a track is stopped because the cell is considered dead (since dead cells can also be tracked). If the tracks are interrupted at the moment when a cell can be visually declared dead, then it raises the question of which cells are actually being considered: perhaps only the fraction of cells that have not yet died but are about to die, making them particularly distinguishable. This might also affect the interpretation of the attention results in the case of apoptosis, where attention appears broadly distributed but, surprisingly (given that discarding 50 frames does not substantially affect classification), tends to have very small values during the last 50 frames. To me, this appears somewhat contradictory and, together with the methodological questions raised above, should be clarified by the authors. I think that using cells with tracks that are comparable (similar starting and ending points, etc.) would provide more clarity.

   - We thank the reviewer for this careful and detailed comment, which raises several points we are happy to clarify.
   - First, regarding when a track is stopped. For the cell-fate prediction task, apoptotic sequences are terminated before the visible event. If the frame of clear morphological collapse is denoted t, we use only frames 0 to $t-1$, i.e. the collapse itself is never part of the input. Consequently, the model never observes the visibly dying or dead cell, and the truncation analysis operated entirely on pre-collapse frames. In particular, at a truncation of 50 frames, the model predicts apoptosis from frames ending approximately 25 h before any visible morphological collapse. We have made the $t-1$ cutoff explicit in the revised Sec. 1.1 (line 123-126). We also show an example apoptotic sequence in Fig. S8 where the $t-1$ frame is included.

- Additionally, the final frames of apoptotic sequences do not receive low attention. They retain relatively high weights but the attention is broadly distributed and partly redundant across frames. For this reason, removing the final frames still leaves comparable information in the earlier ones, so performance is largely preserved. This contrasts with mitosis, where attention and predictive signal are concentrated in the final frames, so their removal causes a much steeper decline in performance. Broadly distributed attention thus reflects a distributed, redundant apoptotic signal that tolerates truncation, whereas concentrated attention reflects a localized mitotic signal that does not.
- Second, regarding the reviewer's concern that the retained cells might be a particularly distinguishable subset, and their suggestion to use tracks with comparable start and end points: we agree this is worth verifying, since apoptotic sequences are on average longer than mitotic ones (see Fig. S1), and progressive truncation experiment relies on more apoptotic sequences. We repeated the truncation analysis on a length-matched subset in which apoptotic and mitotic sequences share the same length distribution and all sequences are at least 50 frames long, so that a comparable population of cells is evaluated at every truncation level (see Fig. S3, line 347-358). On this matched subset, apoptosis recall remained high across the full truncation range (0.91 without truncation to 0.81 at 50 truncated frames), mirroring the results on the full test set. This confirms that the sustained predictability of apoptosis is not an artifact of the length asymmetry between classes or of a particular selected subset, but reflects genuine predictive information present well before the visible death event.

5. A clarification of when the cells are declared as dividing would also be important: there the error of keeping on tracking a cell that is not alive is not present, so the risk of bias is smaller; however, the selection of the endpoint might affect the changes in p53 levels (since nuclear rupture would typically be preceded by a spike in nuclear accumulation).

   - Similar to apoptosis, mitotic sequences are terminated before the visible event. If t denotes the first frame in which two separate daughter cells are visible, we use only frames 0 to $t-1$, so the completed-division frame is excluded from the input. We have made this explicit in the revised Methods (line 123-126).

6. From a biological point of view, there are two aspects that require additional consideration. First, I found it very surprising that mitosis is identified with almost no relation to cell-cycle progression. Could this be related to the fact that, upon chemotherapy, the cell-cycle machinery is perturbed and this might interfere with the reporter? The authors also claim a role for circadian progression, although the circadian signal does not appear to be strongly enriched in high-attention frames according to Figure 7.

   - Regarding the limited role of the cell-cycle channel in mitosis prediction, the cell-cycle reporter is CFP-hGeminin, which is expressed throughout S/G2/M phases and is not mitosis-specific. Given a cell-cycle length of approximately 24 hours, the reporter is expressed at near-constant levels over more than half of the cycle. Moreover, in the last frame before mitosis cells are either in late G2 or early mitosis. Since the reporter signal drops during the metaphase-to-anaphase transition, once the APC complex becomes active, the characteristic cell-cycle change associated with mitosis falls outside our analysis window. We included this into our discussion (line 621-626).
   - Regarding the circadian signal, we agree with the reviewer. As shown in Fig. 7, the circadian channel only exhibits only a small positive effect in high-attention frames of apoptotic sequences, considerably weaker than that of p53. We agree this does not justify the prominence we originally attributed to circadian dynamics in the discussion. We have therefore removed that interpretation from the discussion.

7. As an overall comment, the graphical style adopted by the authors is quite parsimonious. To improve the readability of the manuscript, a larger number of illustrative panels would be beneficial. In particular, illustrating the results with representative frames from time-lapse videos would make the findings much more convincing.

   - Similar to initial Fig. 6, we have added an example of an apoptotic sequence with representative frames (see Fig. S8).
