# Supplementary material for: Understanding cell fate decisions with temporal attention
Source: PLoS Comput Biol. 2026 Oct 12;22(10):e1014813. doi: 10.1371/journal.pcbi.1014813 (PMC13657290; doi:10.1371/journal.pcbi.1014813)
Supplement: Peer review history

# Peer review: Decision Letter 0

**DOI:** 10.1371/journal.pcbi.1014813.r001

**Authors:** Dimitrios Vavylonis, Jianfeng Cao

15 Jun 2026

Understanding cell fate decisions with temporal attention

PLOS Computational Biology

Dear Dr. Bürger,

Thank you for submitting your manuscript to PLOS Computational Biology. After careful consideration, we feel that it has merit but does not fully meet PLOS Computational Biology's publication criteria as it currently stands. Therefore, we invite you to submit a revised version of the manuscript that addresses the points raised during the review process.

Please submit your revised manuscript by Aug 15 2026 11:59PM. If you will need more time than this to complete your revisions, please reply to this message or contact the journal office at ploscompbiol@plos.org. When you're ready to submit your revision, log on to https://www.editorialmanager.com/pcompbiol/ and select the 'Submissions Needing Revision' folder to locate your manuscript file.

Please include the following items when submitting your revised manuscript:

* A letter that responds to each point raised by the editor and reviewer(s). You should upload this letter as a separate file labeled 'Response to Reviewers'. This file does not need to include responses to formatting updates and technical items listed in the 'Journal Requirements' section below.

* A marked-up copy of your manuscript that highlights changes made to the original version. You should upload this as a separate file labeled 'Revised Manuscript with Track Changes'.

* An unmarked version of your revised paper without tracked changes. You should upload this as a separate file labeled 'Manuscript'.

We look forward to receiving your revised manuscript.

Kind regards,

Jianfeng Cao, Ph.D.

Academic Editor

PLOS Computational Biology

Dimitrios Vavylonis

Section Editor

PLOS Computational Biology

**Additional Editor Comments (if provided):**

As discussed by all reviewers, this work is well-motivated and interesting, especially regarding the extensive analysis on temporal information. However, reviewers raise concerns about the details on methodology and experimental results for supporting the conclusion. For the lack of open-source code and data, the reproducibility of this work is also limited for future work. We require authors to conduct substantial revision according to the comments from all reviewers.

**Journal Requirements:**

1) Please ensure that the CRediT author contributions listed for every co-author are completed accurately and in full.

At this stage, the following Authors/Authors require contributions: Florian Bürger, Martim Dias Gomes, Adrián Enrique Granada, Noémie Moreau, and Katarzyna Bozek. Please ensure that the full contributions of each author are acknowledged in the "Add/Edit/Remove Authors" section of our submission form.

The list of CRediT author contributions may be found here: https://journals.plos.org/ploscompbiol/s/authorship#loc-author-contributions

2) We ask that a manuscript source file is provided at Revision. Please upload your manuscript file as a .doc, .docx, .rtf or .tex. If you are providing a .tex file, please upload it under the item type u2018LaTeX Source Fileu2019 and leave your .pdf version as the item type u2018Manuscriptu2019.

3) Please amend your detailed Financial Disclosure statement. This is published with the article. It must therefore be completed in full sentences and contain the exact wording you wish to be published.

1) State what role the funders took in the study. If the funders had no role in your study, please state: "The funders had no role in study design, data collection and analysis, decision to publish, or preparation of the manuscript."

2) If any authors received a salary from any of your funders, please state which authors and which funders..

**Reviewers' comments:**

Reviewer's Responses to Questions

**Comments to the Authors:**

**Please note here if the review is uploaded as an attachment.**

Reviewer #1: In this manuscript, authors applied a deep learning based computational approach to model the longitudinal series of single-cell images in different settings: control versus cisplatin treatment. With the goal of predicting mitosis against apoptosis, authors discussed the effectiveness of their modeling approach and further analyzed important contributors identified by the approach. Overall the concept and study design are very clear, but the datasets, methods, and interpretability analysis would need some further clarification and justifications. Please see below a full list of major and minor concerns:

Major:

- The dataset used for the study could be further clarified:

- In total 51k sequences are curated, out of which only ~21.5k are annotated. Are the remaining trajectories used in any form during the training?

- How are the trajectories labeled? The quantity here seems a bit unrealistic for human labeling, may authors disclose how the labels are acquired? Also is the task a binary classification?

- There is no example image/video in the figures, I would recommend include some example cases.

- From what I understand, the dataset is cell culture, and the cell might migrate in the well. How did authors track the cells across timepoints? Would there be basic metrics to show that this step is done properly? And how would tracking errors propagate to downstream modelings of cell fate?

- Does the dataset contain bright field images? From examples in Fig. 6, it seems that morphology beyond just cell shape is not well captured by these fluorescence channels.

- The modeling choice: CNN for embeddings + ViT on sequence of embeddings would need some justification:

- Why is a ResNet model used and trained from scratch in this project. Given the model size, there could be a high chance of overfitting. Using some kind of pre-trained models for cellular imaging seems more robust.

- It would be preferable to show at least some comparison against naive baselines, such as heuristic morphology (cell size, aspect ratio, etc.) based on segmentation, averaged across the sequence or to replace the CNN embeddings.

- Are the paddings for shorter sequences before or after the sequence? Since positional encoding is used here to indicate timestamp. It makes sense to label the event time (for mitosis/apoptosis) uniformly across sequence, so the "positions" here are more like T-1, T-2, etc. Can authors disclose some more details on the calculation of positional encodings and paddings?

- Are the surrounding cells in the image patches masked? If not, would they affect/confound the model predictions? For example, cells in densely populated area are more likely to undergo apoptosis/mitosis.

- Are distributions of sequence lengths different between mitosis and apoptosis? And between control / cisplatin-treated samples, would there be differences in class balance, distributions of sequence lengths, average morphology, etc.?

- The interpretability part seems to be a highlight of the manuscript, but some aspects could be further polished:

- Fig. 5 shows some features captured in the "important" frames, can we further examine their directionalities towards the prediction of mitosis/apoptosis? For example, does high p53 mean higher chance of apoptosis?

- Since each sequence contains longitudinal data points, it is expected that frame t might contain lots of similar information as frame (t-1) and (t+1). The strategy of using attention weights as feature importance might not be able to natively capture this longitudinal continuity, may authors comment on their choice here?

- It is also widely recognized that attention weights could be arbitrary. In addition to this attention-based importance scores, would there be alternative measures to get frame importance? A naive idea would be to mask certain frames (just like what authors did in Fig. 3) and see how such masking lead to changes in predictions.

- Since the key topic of this manuscript is about cell fate, I think a key type of discovery is some kind of trigger events detected by the sequence transformer that heavily affect the predictions of cell fate. Authors have only discussed these qualitatively ("rounding of the cell and fluctuation of nuclear fluorescence signals"), but I would recommend extending the masking experiments in Fig. 3 to further reveal this type of information. For example, given a sequence, can we show the curve of predicted mitosis/apoptosis along the sequence, where each point's prediction is only based on the history observed until this point. A sharp change in this curve could be indicative of a trigger event.

- Would there be any differences between control / cisplatin-treated cases in terms of their interpretability results? Given the effect of cisplatin as a chemotherapy reagent to trigger apoptosis, it can be imagined that the important frames might show different distributions as compared to control cases. Discussion on such differences is missing.

- In practice, it would be nearly impossible to get continuous measurements out of a real cancer patient's tumor. In cases where only snapshots can be acquired (high-dimensional spatial omics, for example), may authors discuss how this proposed modeling approach/concept can be applied or adapted?

Minor:

- Fig. 4 is a bit hard to read, maybe use a scatter/line plot instead.

- Line 170-174: "Across all sequences, this results in n p-values and n corresponding Cliff’s delta values ..." What is `n` here? Do you mean "across ONE sequence" or actually across all sequences of the dataset? I find this paragraph a bit hard to understand.

Reviewer #2: Overall assessment:

Bürger et al., has developed a Transformer-based model that predicts single-cell fate directly from raw time-lapse microscopy sequences of cisplatin-treated cancer cells, reporting a balanced accuracy of 0.94. They introduce an explainability pipeline that finds high attentionframes and by using a Cliff's-delta comparison they investigate which morphological or intensity-based features leads to the high attention frames. Here they find that the p53 channel showed a pronounced positive effect.

In my opinion, the problem is well-motivated and investigates a very deep question. The main results and conclusions are very interesting though vital aspects remain unclear. The dataset applied for this analysis is small compared to normal ML approaches (which is not a bad thing) but good enough to draw conclusions. The writing of the paper is very direct (which again is not a bad thing) for the most part clear. Therefore I do see that this work can be published in Plos comp. Biol, however I have however a series of questions and points where I believe the authors do not support their main conclusions and to publish this paper would therefore require a major revision.

General problem:

The authors report very profound results but I believe the evidence of these are often thin. First of all, the two central claims of the paper: 10 h predictability and importance of p53 channel is not supported directly by a figure. I really need the authors to make this more clear.

Major points:

A) Figures: I would very much like more figures showing the dynamical traces of many of these observables - in particular p53 and circadian information. Also I need figures (histograms at least) that outline what makes the high attention weights dominating (see my point E on this also). Also, there should be a qualitative apoptotic example analogous to mitosis in Fig. 6. Furthermore, Fig. 4 is really hard to read quantitatively; the numbers quoted in text (0.62, 0.22, 0.88, 0.42) deserve a line plot with confidence bands, not a grayscale strip.

B) Regarding the 10 h claim, then I cannot trace it to a specific quantitative result. In Fig 3a, the authors show BAcc stays above approximately 0.9 up to 20 frames removed (which means 10 h) - but this definition should be more clearly stated. Also, this is overall BAcc on a set dominated by mitosis, and Fig. 3c shows mitotic recall already drops at 5 frames. I therefore understand this as the 10 h statement holds mainly for apoptosis, not as a general claim? Please describe this result in greater detail and state the claim per-class, define it against a stated baseline, and mark the relevant threshold explicitly on Fig. 3.

C) I find the p53 claim very interesting but was disappointed with the (lack of) evidence revealed in the paper. In the discussion, the authors discuss how: “the distinct temporal attention profiles for mitotic versus apoptotic sequences mirror the distinct p53 dynamical states that mechanistic studies have identified as fate-determining”, citing work of the Lahav lab. However, the authors show no traces of neither p53 or circadian information and in the main text they write that high-attention frames have elevated p53 intensity on average. There is no analysis of p53 dynamics despite the authors explicitly invoking these as the mechanism. I therefore see two options: either add an analysis that directly tests a dynamical hypothesis based on p53 trajectories and ask whether a dynamical feature (pulse count, slope before event) separates fates and aligns with high-attention frames or substantially downtune the claims of mechanisms and explainability claims throughout the text. As written, the interpretation is largely overstated compared to the evidence revealed.

D) A main claim of the paper is that temporal attention matters and to validate this the authors should perform a non-temporal baseline. In particular Fig. 3d shows mitosis can be estimated approximately 0.94 recall from the last 5 frames alone - this could suggest that a static last-frame classifier might already do well for mitosis? This needs to be thoroughly discussed.

E) The second half of the paper (Sec. 2.2 and the p53/circadian/morphology interpretation in the Discussion) rests on a critical assumption: that the frames receiving high attention weights are the frames the model actually relies on for its prediction. I am no expert in the current developments in ML, but it is my impression that based on papers by (Serrano, S., & Smith, N. A. (2019). Is attention interpretable? and Jain, S., & Wallace, B. C. (2019). Attention is not explanation - and counter argument Wiegreffe, S., & Pinter, Y. (2019). Attention is not not explanation) it seems to me that this could be validated even further. My simple suggestion for the authors is to show a figure where you take the correctly classified sequences and measure how much prediction degrades when you remove frames, comparing three conditions: 1) masking the high-attention frames, 2) masking the same number of randomly chosen frames and 3) masking the same number of low-attention frames. If attention is faithful, masking H_s should hurt prediction substantially more than masking random or low-attention frames. If the three are comparable, the attention weights are not tracking importance. In my opinion this would strengthen the central claim of this paper.

Minor points:

a) The reported embedding dimension is "2024-dimensional" (line 129). Do you mean 2048 for ResNet-50?

b) BAcc on the cisplatin-only test set is 0.958 (line 243) vs. 0.94 on the full test set (line 215). Please clarify which is the main number and why they differ.

c) Cisplatin was given at three dose levels (line 101). Are temporal/attention patterns dose-dependent? Pooling across doses may mask heterogeneity relevant to the overall fate claim. At minimum, report the dose composition of the apoptotic vs. mitotic sets.

Reviewer #3: In this paper, the authors analyze an existing live-cell imaging dataset obtained upon treatment with cisplatin, a common chemotherapeutic agent, in which treated cells, followed for hours, were annotated as mitotic or apoptotic. In this dataset, nuclei, p53 levels, cell-cycle progression, and circadian progression can be monitored through the use of appropriate reporters. By applying a transformer model, the authors are able to accurately classify mitotic and apoptotic cells. Furthermore, attention and masking allow them to identify the variables that contribute most to the classification, with the potential to provide insights into the biological determinants of cell fate decisions.

Using machine-learning methods able to extract features and potentially biological insights into what determines cellular outcomes from live-cell imaging data can certainly be very useful. At the same time, a careful evaluation of the precise advantages and disadvantages of each methodology, and of the extent to which the systems used can really provide biological insight, is necessary. At this stage, this paper does not address these two important issues in a convincing way. To do so, several points must be addressed so that the contribution and value of the present work become evident.

1 The authors declare that their dataset comes from a system developed by some of them called “TransientTrack” (https://arxiv.org/html/2512.01885v1). Inspecting this work, it is evident that it is a machine-learning-based system that is also able to track and classify cells. At this point, it is not clear to me whether the annotations used in the present study were generated with this method. Furthermore, it would be important to understand how the classification performed here compares to that performed by TransientTrack (which is only possible if both are assessed against an independent manually annotated dataset). More details on how the annotations were generated would be necessary to properly assess the biological potential of the method and to ensure that there are no experimental biases.

2. A method able to automatically classify cells undergoing apoptosis, mitosis, or neither of these two outcomes would be of great interest, given the growing availability of live-cell imaging experiments and datasets. It would therefore have been worthwhile to test whether the proposed method was able to classify cells into these three classes: apoptotic, mitotic, or neither. Such a classification should also be compared against TransientTrack.

3. A major hurdle in convincing the reader of the power of the method is that it is validated on only one experimental condition: cells treated with cisplatin. Applying it at least to untreated versus treated cells would have provided more convincing evidence of both the validity of the method and the biological insights it can provide. For example, some evidence points to a role of p53 accumulation in cell death (PMID: 27062928) and to p53 bursts during the cell cycle (PMID: 20598361). I would expect these behaviors to change and produce different patterns in treated cells compared to untreated cells. This aspect is unfortunately unexplored.

4. The classification results shown in Figure 4 are striking and deserve further comment. From the results, it appears that cells treated with cisplatin are predicted to be apoptotic very accurately even when the last 50 frames are removed. This roughly corresponds to 25 hours, which is typically the time required for cells to die upon cisplatin treatment according to PMID: 27062928. A point that the authors need to clarify is the exact moment at which a track is stopped because the cell is considered dead (since dead cells can also be tracked). If the tracks are interrupted at the moment when a cell can be visually declared dead, then it raises the question of which cells are actually being considered: perhaps only the fraction of cells that have not yet died but are about to die, making them particularly distinguishable. This might also affect the interpretation of the attention results in the case of apoptosis, where attention appears broadly distributed but, surprisingly (given that discarding 50 frames does not substantially affect classification), tends to have very small values during the last 50 frames. To me, this appears somewhat contradictory and, together with the methodological questions raised above, should be clarified by the authors. I think that using cells with tracks that are comparable (similar starting and ending points, etc.) would provide more clarity.

5. A clarification of when the cells are declared as dividing would also be important: there the error of keeping on tracking a cell that is not alive is not present, so the risk of bias is smaller; however, the selection of the endpoint might affect the changes in p53 levels (since nuclear rupture would typically be preceded by a spike in nuclear accumulation).

6. From a biological point of view, there are two aspects that require additional consideration. First, I found it very surprising that mitosis is identified with almost no relation to cell-cycle progression. Could this be related to the fact that, upon chemotherapy, the cell-cycle machinery is perturbed and this might interfere with the reporter? The authors also claim a role for circadian progression, although the circadian signal does not appear to be strongly enriched in high-attention frames according to Figure 5.

7. As an overall comment, the graphical style adopted by the authors is quite parsimonious. To improve the readability of the manuscript, a larger number of illustrative panels would be beneficial. In particular, illustrating the results with representative frames from time-lapse videos would make the findings much more convincing.

**********

**Have the authors made all data and (if applicable) computational code underlying the findings in their manuscript fully available?**

The PLOS Data policy requires authors to make all data and code underlying the findings described in their manuscript fully available without restriction, with rare exception (please refer to the Data Availability Statement in the manuscript PDF file). The data and code should be provided as part of the manuscript or its supporting information, or deposited to a public repository. For example, in addition to summary statistics, the data points behind means, medians and variance measures should be available. If there are restrictions on publicly sharing data or code —e.g. participant privacy or use of data from a third party—those must be specified.

Reviewer #1: Yes

Reviewer #2: None

Reviewer #3: **No:**  I could not find data nor clear access to imaging data nor the code - but this could be my mistake.

**********

PLOS authors have the option to publish the peer review history of their article (what does this mean?). If published, this will include your full peer review and any attached files.

If you choose “no”, your identity will remain anonymous but your review may still be made public.

**Do you want your identity to be public for this peer review?**  For information about this choice, including consent withdrawal, please see our Privacy Policy.

Reviewer #1: **Yes:**  Zhenqin Wu

Reviewer #2: **Yes:**  Mathias Heltberg

Reviewer #3: No

[NOTE: If reviewer comments were submitted as an attachment file, they will be attached to this email and accessible via the submission site. Please log into your account, locate the manuscript record, and check for the action link "View Attachments". If this link does not appear, there are no attachment files.]

**Figure resubmission:**

After uploading your figures to PLOS’s NAAS tool - https://ngplosjournals.pagemajik.ai/artanalysis, NAAS will process the files provided and display the results in the "Uploaded Files" section of the page as the processing is complete. If the uploaded figures meet our requirements (or NAAS is able to fix the files to meet our requirements), the figure will be marked as "fixed" above. If NAAS is unable to fix the files, a red "failed" label will appear above. When NAAS has confirmed that the figure files meet our requirements, please download the file via the download option, and include these NAAS processed figure files when submitting your revised manuscript.

**Reproducibility:**

To enhance the reproducibility of your results, we recommend that authors of applicable studies deposit laboratory protocols in protocols.io, where a protocol can be assigned its own identifier (DOI) such that it can be cited independently in the future. Additionally, PLOS ONE offers an option to publish peer-reviewed clinical study protocols. Read more information on sharing protocols at https://plos.org/protocols?utm_medium=editorial-email&utm_source=authorletters&utm_campaign=protocols

# Author comment: Author response to Decision Letter 1

**DOI:** 10.1371/journal.pcbi.1014813.r002

5 Aug 2026

Attachment Submitted filename: Response to Reviewers.pdf

# Peer review: Decision Letter 1

**DOI:** 10.1371/journal.pcbi.1014813.r003

**Authors:** Dimitrios Vavylonis, Jianfeng Cao

10 Sep 2026

Dear Mr. Bürger,

We are pleased to inform you that your manuscript 'Understanding cell fate decisions with temporal attention' has been provisionally accepted for publication in PLOS Computational Biology.

Before your manuscript can be formally accepted you will need to complete some formatting changes, which you will receive in a follow up email. A member of our team will be in touch with a set of requests.

Please note that your manuscript will not be scheduled for publication until you have made the required changes, so a swift response is appreciated.

IMPORTANT: The editorial review process is now complete. PLOS will only permit corrections to spelling, formatting or significant scientific errors from this point onwards. Requests for major changes, or any which affect the scientific understanding of your work, will cause delays to the publication date of your manuscript.

Should you, your institution's press office or the journal office choose to press release your paper, you will automatically be opted out of early publication. We ask that you notify us now if you or your institution is planning to press release the article. All press must be co-ordinated with PLOS.

Thank you again for supporting Open Access publishing; we are looking forward to publishing your work in PLOS Computational Biology.

Best regards,

Jianfeng Cao, Ph.D.

Academic Editor

PLOS Computational Biology

Dimitrios Vavylonis

Section Editor

PLOS Computational Biology

***********************************************************

According to the feedback from all three reviewers, we are pleased to accept this work for publication in PLOS Computational Biology. Please address the minor concerns raised by the reviewers.

Reviewer's Responses to Questions

**Comments to the Authors:**

**Please note here if the review is uploaded as an attachment.**

Reviewer #1: The authors have resolved all my concerns, I have no further questions or comments.

Reviewer #2: I think this is a really great work and I am very pleased with the way the authors have addressed my concerns. A small note is that the abstract still read 10h - I guess this should be 8 h?

Reviewer #3: The authors have addressed the concerns that I raised. I also found that the work following the other reviewers' comments has improved the work.

The only recommendation that was not followed has to do with the limited amount of data shown in the main figures. Related with this, I think that the additional analysis performed (e.g. Figs S6 and S7) are of interest - as evidenced also by the comparatively high fraction of the text devoted to it- so I still think that at least part of these figure panels could be "promoted" to main figures. This is just a recommendation.

However, this is more a stylistic decision for the authors (and maybe for the Editor) so I'd recommend acceptance independently of the decision they will make.

**********

**Have the authors made all data and (if applicable) computational code underlying the findings in their manuscript fully available?**

The PLOS Data policy requires authors to make all data and code underlying the findings described in their manuscript fully available without restriction, with rare exception (please refer to the Data Availability Statement in the manuscript PDF file). The data and code should be provided as part of the manuscript or its supporting information, or deposited to a public repository. For example, in addition to summary statistics, the data points behind means, medians and variance measures should be available. If there are restrictions on publicly sharing data or code —e.g. participant privacy or use of data from a third party—those must be specified.

Reviewer #1: Yes

Reviewer #2: None

Reviewer #3: Yes

**********

PLOS authors have the option to publish the peer review history of their article (what does this mean?). If published, this will include your full peer review and any attached files.

If you choose “no”, your identity will remain anonymous but your review may still be made public.

**Do you want your identity to be public for this peer review?**  For information about this choice, including consent withdrawal, please see our Privacy Policy.

Reviewer #1: **Yes:**  Zhenqin Wu

Reviewer #2: **Yes:**  Mathias Heltberg

Reviewer #3: No

# Editor report: Acceptance letter

**DOI:** 10.1371/journal.pcbi.1014813.r004

**Authors:** Dimitrios Vavylonis, Jianfeng Cao

PCOMPBIOL-D-26-00871R1

Understanding cell fate decisions with temporal attention

Dear Dr Bürger,

I am pleased to inform you that your manuscript has been formally accepted for publication in PLOS Computational Biology. Your manuscript is now with our production department and you will be notified of the publication date in due course.

The corresponding author will soon be receiving a typeset proof for review, to ensure errors have not been introduced during production. Please review the PDF proof of your manuscript carefully, as this is the last chance to correct any errors. Please note that major changes, or those which affect the scientific understanding of the work, will likely cause delays to the publication date of your manuscript.

Soon after your final files are uploaded, unless you have opted out, the early version of your manuscript will be published online. The date of the early version will be your article's publication date. The final article will be published to the same URL, and all versions of the paper will be accessible to readers.

For Research, Software, and Methods articles, you will receive an invoice from PLOS for your publication fee after your manuscript has reached the completed accept phase. If you receive an email requesting payment before acceptance or for any other service, this may be a phishing scheme. Learn how to identify phishing emails and protect your accounts at https://explore.plos.org/phishing.

Thank you again for supporting PLOS Computational Biology and open-access publishing. We are looking forward to publishing your work!

With kind regards,

Sharmila Kamatchi

PLOS Computational Biology | Carlyle House, Carlyle Road, Cambridge CB4 3DN | United Kingdom ploscompbiol@plos.org | Phone +44 (0) 1223-442824 | ploscompbiol.org | @PLOSCompBiol
